# Quantitative sequencing using BID-seq uncovers abundant pseudouridines in mammalian mRNA at base resolution

**DOI:** 10.1038/s41587-022-01505-w

**Published:** 2022-10-27

**Authors:** Qing Dai, Li-Sheng Zhang, Hui-Lung Sun, Kinga Pajdzik, Lei Yang, Chang Ye, Cheng-Wei Ju, Shun Liu, Yuru Wang, Zhong Zheng, Linda Zhang, Bryan T. Harada, Xiaoyang Dou, Iryna Irkliyenko, Xinran Feng, Wen Zhang, Tao Pan, Chuan He

**Affiliations:** 1 Department of Chemistry, The University of Chicago, Chicago, IL USA; 2 Howard Hughes Medical Institute, The University of Chicago, Chicago, IL USA; 3 First Maternity and Infant Hospital, School of Medicine, Tongji University, Shanghai, China; 4 Pritzker School of Molecular Engineering, The University of Chicago, Chicago, IL USA; 5 Department of Human Genetics, The University of Chicago, Chicago, IL USA; 6 Department of Biochemistry and Molecular Biology, The University of Chicago, Chicago, IL USA; 7 Institute for Biophysical Dynamics, The University of Chicago, Chicago, IL USA

**Keywords:** RNA sequencing, RNA

## Abstract

Functional characterization of pseudouridine (Ψ) in mammalian mRNA has been hampered by the lack of a quantitative method that maps Ψ in the whole transcriptome. We report bisulfite-induced deletion sequencing (BID-seq), which uses a bisulfite-mediated reaction to convert pseudouridine stoichiometrically into deletion upon reverse transcription without cytosine deamination. BID-seq enables detection of abundant Ψ sites with stoichiometry information in several human cell lines and 12 different mouse tissues using 10–20 ng input RNA. We uncover consensus sequences for Ψ in mammalian mRNA and assign different ‘writer’ proteins to individual Ψ deposition. Our results reveal a transcript stabilization role of Ψ sites installed by TRUB1 in human cancer cells. We also detect the presence of Ψ within stop codons of mammalian mRNA and confirm the role of Ψ in promoting stop codon readthrough in vivo. BID-seq will enable future investigations of the roles of Ψ in diverse biological processes.

## Abstract (web summary)

Pseudouridine sites in mRNA are detected at base resolution and functionally investigated.

## Main

Posttranscriptional RNA modifications occur in all life forms and all types of RNA^1,2^. Ψ is a prevalent RNA modification that can impact diverse biological functions of different RNA species^3^. Ψ is also known to exist in mRNA in mammals^4–6^. Thirteen putative pseudouridine synthase (PUS) enzymes have been annotated in the human genome^7–9^, and mutations in these enzymes have been associated with a wide range of human diseases^10–12^. Specific PUS enzymes have been reported to catalyze Ψ deposition in mammalian mRNA^13^, which may impact mRNA processing, metabolism and translation. However, mechanistic studies have been hampered by an inability to comprehensively detect Ψ at base resolution and to quantify the modification level or stoichiometry at the modified sites.

Previous detection of Ψ within RNA has relied mostly on its reaction with *N*-cyclohexyl-*N*′-(2-morpholinoethyl)carbodiimide methyl-*p*-toluenesulfonate (CMC) to produce CMC-modified Ψ, which generates a stop signature during reverse transcription (RT)^14^. This approach has been employed for transcriptome-wide Ψ mapping (named ‘Ψ-seq’ or ‘Pseudo-seq’)^4,5^, identifying a modest number of Ψ sites in human mRNA with only 13 sites overlapped between the two independent datasets, accounting for a small proportion of the Ψ sites in human mRNA based on liquid chromatography tandem mass spectrometry (LC-MS/MS)^6^. An azide-modified CMC has been used to enrich Ψ-containing RNA fragments for sequencing (CeU-seq)^6^, allowing the detection of many more Ψ sites; however, it lacks base resolution and stoichiometry information at the modified sites.

Taking advantage of a recently reported reactivity of bisulfite (BS) towards Ψ^15,16^, we report here BS-induced deletion sequencing (BID-seq) as a base-resolution method for quantitative and transcriptome-wide mapping of Ψ. We discovered a BS reaction condition that quantitatively converts Ψ to a Ψ-BS adduct without cytosine deamination, leading to unique deletion signatures at Ψ sites during reverse transcription. We used BID-seq to detect fraction-altered Ψ sites upon knockdown of individual PUS enzymes in HeLa cells, and identified ‘writer’ proteins for Ψ sites in mRNA. We observed more Ψ-modified mRNA sites in mouse tissues than in human cell lines, with highly Ψ-modified transcripts displaying higher abundance and tissue-specific features. We identified TRUB1 as a main mRNA Ψ ‘writer’ protein that regulates mRNA stability. We additionally uncovered a number of Ψ sites within stop codons of mammalian mRNA, and confirmed the role of Ψ in promoting stop codon readthrough^17–19^ in vivo.

## Results

### A new BS condition quantitatively converts Ψ to Ψ-BS adduct

**Fig. 1:**
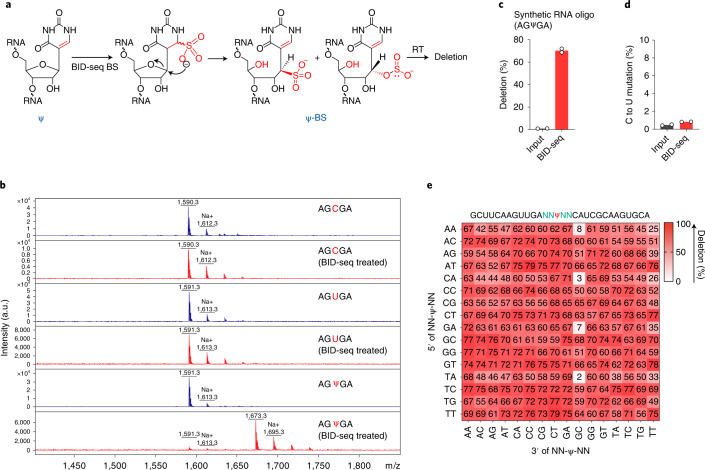
BID-seq quantitatively detects Ψ sites as deletion signatures. **a**, Chemical structure of the Ψ-BS adduct after bisulfite treatment. **b**, BID-seq BS selectively reacts with Ψ and completely converts it into the Ψ-BS adduct under optimized conditions, without affecting normal C or U bases in RNA. **c**, The deletion ratio at the 100% modified Ψ site within the AGΨGA motif (synthesized RNA oligo) after BID-seq treatment versus that in the input. **d**, The average C to U mutation ratio at normal cytidine bases in synthesized RNA oligo after BID-seq treatment versus that in input. For **c**–**d**, *n* = 2 biologically independent samples. **e**, Heatmap plot for deletion ratios on 256 motifs (NNΨNN) after BS treatment in BID-seq, which contain one 100% modified Ψ within each motif. Source data

In a recent effort to map m^5^C in RNA, Khoddami et al. made a surprising observation that BS treatment could lead to modest base deletions during RT at Ψ sites in RNAs (RBS-seq) (Fig. 1a)^15,16^. The formation of a Ψ-BS adduct was shown to be the key intermediate that leads to deletion readout upon reverse transcription^15^. In total, 15 and 20 Ψ sites (deletion rate >5%) were detected in human 18S and 28S rRNA, respectively, using the RBS-seq protocol, but the signals on human mRNA were weak, with only 78 sites detected with a deletion rate of greater than 5%^16^. The conventional BS reaction condition in RBS-seq inevitably converted all the cytosines into uracils and thus reduced read complexity, resulting in a notable proportion of reads that could not be aligned to mRNA exons. Nevertheless, the discovery of Ψ-BS-adduct-induced deletion during RT provided a completely new principle for potential Ψ detection.

Following these intriguing observations^15,16^, we tested two commercial bisulfite kits (Zymo and Epigentek) used for conventional BS treatment on synthetic 5-mer RNA oligonucleotides AGXGA (X = C or Ψ). In both cases, we observed quantitative C-to-U conversion, but no formation of Ψ-BS adducts (Supplementary Fig. 1a). We then examined the published RBS-seq condition to measure the conversion efficiency of Ψ to Ψ-BS adduct^16^. Although matrix-assisted laser desorption/ionization-time of flight (MALDI-TOF) MS showed quantitative C-to-U conversion, the efficiency of Ψ-BS adduct formation varied and was less than 30% among four replicates (Fig. 1a and Supplementary Fig. 1b), explaining the low sensitivity in detection of Ψ using the previous protocol.

It is known that the protonation of N3 in cytosine at acidic pH (around 5.1) is critical to BS-mediated deamination, whereas a neutral pH is more suitable for the BS reaction with uracil^20^. We reasoned that neutral pH would inhibit C-to-U conversion but promote Ψ reaction with BS to yield higher levels of Ψ-BS (Fig. 1a). Indeed, BS treatment of the model RNA probes at neutral pH followed by MALDI-TOF MS revealed quantitative conversion of Ψ to Ψ-BS adduct without any detectable C-to-U conversion (Fig. 1b).

To optimize Ψ detection, we treated a 30-mer Ψ-containing RNA probe (with a AGΨGA motif) with BS at neutral pH (2.4 M Na_2_SO_3_ and 0.36 M NaHSO_3_) and screened commercial reverse transcriptases. We found that SuperScript IV generated a high deletion rate (~70%) at the fully modified Ψ site after the new bisulfite reaction followed by RT, amplification and sequencing, whereas the deletion ratio was almost undetectable (<1%) in the untreated ‘input’ (Fig. 1c). Note that deletion rates of unmodified bases (A, C, G, U) and the C-to-U conversion at C bases were undetectable in both treated and untreated samples (Fig. 1d), indicating very low background and no reduction in read complexity caused by potential cytosine deamination. To examine the deletion rate dependency on the sequence context, we built libraries with 30-mer RNA oligonucleotides containing NNΨNN (N = A or C or G or U) as spike-in and performed BID-seq. We found that 232 out of 256 motifs gave deletion rates over 50% at the Ψ site, with 252 out of 256 motifs displaying deletion rates above 25% (Fig. 1e). After BID-seq, the unmodified probes containing 0% Ψ (NNUNN) displayed deletion ratios of less than 5% for most sequence motifs; high background (around 10–25% deletion ratio) was observed in only a few motifs containing ACΨ-, CUΨ-, GCΨ-, GUΨ- or -ΨUC, -ΨUG (Supplementary Fig. 1c). When calling Ψ candidate sites in biological samples, we set the deletion rate at greater than 1.5-fold over the background at each candidate site to eliminate potential false positives arising from the background in our analysis pipeline.

Together, we show that BID-seq quantitatively converts Ψ to the Ψ-BS adduct without detectable C-to-U conversion, and that SuperScript IV generates high deletion rates at the BS-modified Ψ sites in most sequence contexts during RT, confirming that BID-seq is highly sensitive and specific for Ψ detection. With spike-in probes containing varied Ψ levels to calibrate sequence-context-dependent deletion rate^21^, we can further calculate the stoichiometry at the Ψ-modified sites.

### Validation of BID-seq

**Fig. 2:**
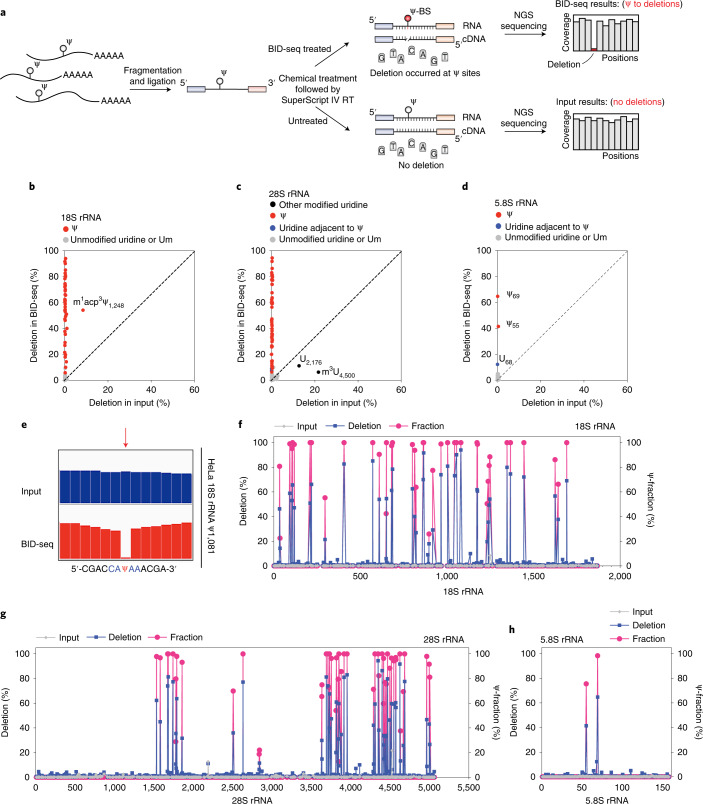
BID-seq detects known Ψ sites in human ribosomal RNA with modification stoichiometry. **a**, Flowchart of library construction pipeline for BID-seq, revealing Ψ modification fraction by deletion ratio signature. **b**, Two-dimensional (2D) plot for deletion ratios of known Ψ sites in HeLa 18S ribosomal RNA, in BID-seq treated library versus input. **c**, 2D plot for deletion ratios of known Ψ sites in HeLa 28S ribosomal RNA, in BID-seq treated library versus input. **d**, 2D plot for deletion ratios of known Ψ sites in HeLa 5.8S ribosomal RNA, in BID-seq treated library versus input. **e**, An example IGV plot of the highly modified Ψ site at position 1,081 of HeLa 18S ribosomal RNA, within a CAΨAA motif. **f**–**h**, Deletion and Ψ fraction detected by BID-seq in HeLa 18S rRNA (**f**), 28S rRNA (**g**), and 5.8S rRNA (**h**), respectively. After BS treatment in BID-seq, the deletion rates and Ψ fractions are marked in blue and pink, respectively. Source data

To validate BID-seq in biological samples, we developed a BID-seq protocol to map Ψ in various RNA species from biological samples (Fig. 2a). We first applied BID-seq to validate Ψ detection in rRNA from HeLa cells. To identify notable Ψ deletion signatures, we set the Ψ detection criteria as follows: (1) deletion rate above 5% (with deletion count above five in BID-seq libraries); (2) deletion rate below 1% in ‘Input’ libraries; (3) total reads coverage depth above 20 in both BID-seq and ‘Input’ libraries; (4) deletion rate above 1.5-fold over background in any given sequence motif (defined as the deletion rates detected from RNA probes containing 0% Ψ, as in Supplementary Fig. 1c). In addition, we excluded sites that tend to be false positives, specifically uracil sites at the neighboring nucleotide 3′ or 5′ to the known Ψ sites.

Applying all these criteria for Ψ detection, we identified 42, 53 and 2 known Ψ sites in HeLa 18S, 28S and 5.8 rRNAs^22^, respectively, without any false positives; these known Ψ sites all exhibited notable deletion rates ranging from 5% to 95% in BID-seq (Fig. 2b–d). A representative highly modified Ψ1,081 site in HeLa 18S rRNA is visualized in an original Integrative Genomics Viewer (IGV) plot (Fig. 2e). Notably, the deletion rates at these Ψ sites in untreated ‘input’ were less than 1%, except for a couple of known modifications such as m^1^acp^3^Ψ_1,248_ at 18S rRNA^23^, m^3^U_4,500_ at 28S rRNA and an interesting uncharacterized U_2,176_ site at 28S rRNA (Fig. 2b,c).

To quantify the modification fraction at each Ψ site by deletion rate, we mixed oligo probes containing NNΨNN and NNUNN (with different stoichiometry of Ψ) as controls to plot calibration curves for these sequence contexts (Supplementary Fig. 2a and Table 1). The high mutation rates on 232 motifs, low background for most of these motif contexts and the approximately hyperbola calibration curves in BID-seq enabled sensitive detection of Ψ as well as estimation of Ψ stoichiometry. Based on the calibration curves, the fractions of these Ψ sites in HeLa 18S, 28S and 5.8S rRNAs were calculated to be around 20–100%, generally consistent with those measured by mass spectrometry^22^ (Fig. 2f–h and Supplementary Tables 2–4). Among 43 and 61 known Ψ sites uncovered by mass spectrometry in HeLa 18S and 28S rRNAs^22^, respectively, 9 sites were not detected by BID-seq for three reasons: (1) low modification fraction: 18S rRNA Ψ1,136 and 28S rRNA Ψ4,463 (Supplementary Fig. 2b–e); (2) no reads coverage at the Ψ site because of dramatic RT stop caused by multiple highly modified Ψ sites within a narrow region: 28S rRNA Ψ3,741/Ψ3,743/Ψ3,747/Ψ3,749 and 28S rRNA Ψ4,266/Ψ4,269 (Supplementary Fig. 2d–f); (3) m^3^U adjacent to a Ψ site that seems to interfere with the BS reaction on the Ψ base or the subsequent RT: 28S rRNA Ψ4,501 (Supplementary Fig. 2d–f). These represent potential limitations of BID-seq in mapping Ψ sites.

Compared with BID-seq, RBS-seq detected 15 and 20 Ψ sites in 18S and 28S rRNA, respectively, because of low deletion rates, with deletion rates close to zero for other known Ψ sites (Supplementary Fig. 2g,h). We also applied BID-seq to small RNAs (<200 nt) from HeLa cells, and validated highly modified Ψ sites in both H/ACA box and C/D box snoRNAs (Supplementary Fig. 2i,j), including snoRNA Ψ sites previously revealed by Ψ-seq^5^.

### BID-seq maps Ψ in mRNA from human cell lines

**Fig. 3:**
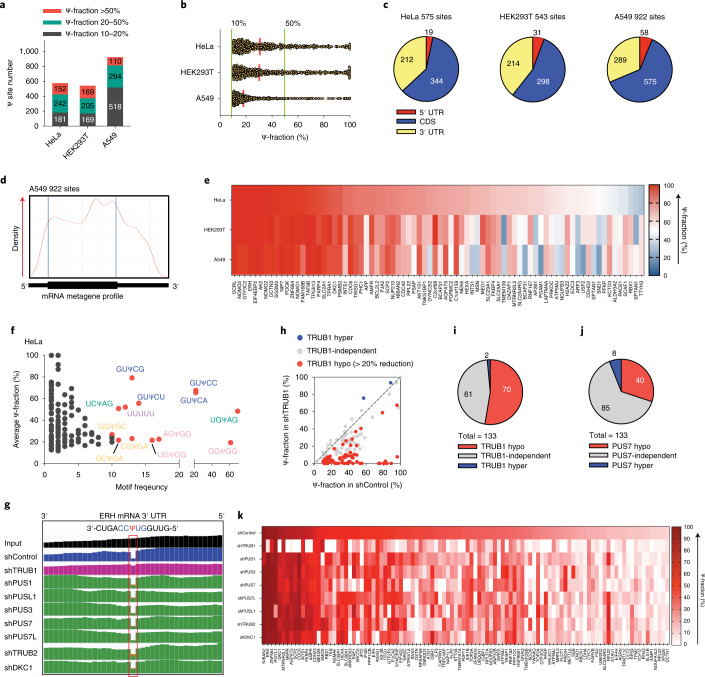
BID-seq detects Ψ sites at base resolution in human mRNA and characterizes the ‘writer’ protein for individual Ψ sites. **a**, BID-seq reveals 575, 543 and 922 Ψ sites (modification fraction above 10%) in HeLa, HEK293T and A549 cells, respectively. **b**, Modification level distribution of Ψ sites in mRNA from HeLa, HEK293T and A549 cells, with the definition of highly modified Ψ sites as those above 50% Ψ-fraction. **c**, Pie chart showing the distribution of mRNA Ψ sites in HeLa, HEK293T and A549 cells, with stoichiometry ≥10% in three mRNA segments. **d**, Metagene plot of 922 Ψ sites (modification fraction >10%) in A549 mRNA. **e**, Heatmap plot of Ψ-fraction for 78 overlapped Ψ sites with above 50% Ψ-fraction in at least one human cell line and above 10% Ψ-fraction in three cell lines, in a matrix of the corresponding gene name versus each cell line. **f**, Distribution of motifs for 575 Ψ sites in HeLa mRNA, with ‘*x* axis’ as the motif frequency and ‘*y* axis’ showing the average Ψ modification fraction of each motif. **g**, Example IGV plot to show raw reads coverage at the highly modified Ψ site in HeLa *ERH* mRNA. The deletion signatures reflect the modification level change in shTRUB1 versus shControl, but not depletion of other PUS enzymes. **h**, Among 133 Ψ sites (above 10% Ψ-fraction) in shControl HeLa mRNA, scatter plot of BID-seq data shows the reduced Ψ-fraction at 70 Ψ sites in TRUB1-depleted cells. **i**, Pie chart of TRUB1 hypo-regulated, hyper-regulated and TRUB1-independent Ψ sites. **j**, Pie chart of PUS7 hypo-regulated, hyper-regulated and PUS7-independent Ψ sites. **k**, Heatmap plot of Ψ-fraction for 104 Ψ sites that show reduced modification level under the depletion of a specific PUS enzyme or multiple PUS enzymes, in a matrix of the corresponding gene name versus the knockdown of each PUS enzyme. Source data

We optimized BID-seq to be compatible with low RNA input^21,24^, and then applied it to 10–20 ng polyA-tailed RNA from HeLa, HEK293T and A549 cells. In addition to the aforementioned criteria for Ψ detection, we added one more Ψ modification fraction cutoff and focused on mRNA sites >10% Ψ stoichiometry, as the candidate sites. We identified 575, 543 and 922 Ψ sites in mRNA from HeLa, HEK293T and A549 cells, respectively (Fig. 3a), which all showed clear internal deletion signatures (Supplementary Fig. 3a and Tables 5–7). Meanwhile, we set up an additional cutoff criterion that requires a deletion count of more than ten to assign hundreds of ‘confident’ Ψ sites in human mRNA (Supplementary Fig. 3b and Tables 5–7). Most of these mRNA Ψ sites display the modification fraction at 10–40% (Supplementary Fig. 3c), but we also identified 152, 169 and 110 highly modified mRNA Ψ sites (>50% Ψ fraction) in the three human cell lines (Fig. 3a), with a continuous distribution of Ψ fraction from 50% all the way to close to 100% (Fig. 3b). The mRNA Ψ sites distribute mostly in coding sequence (CDS) and 3′-UTR (Fig. 3c), similar to the distribution pattern observed previously using CeU-seq^6^. In the metagene profile, an example of the mRNA Ψ candidate sites in A549 cells shows accumulation in the CDS region (Fig. 3d). The common gene ontology (GO) clusters of HeLa and A549 cells enrich the functions such as microtubule/cytoskeleton, ribosome, membrane, actin binding, ATP binding, translation, mRNA processing, etc. (Supplementary Fig. 3d). Note that Ψ can be either shared or cell-line specific. We uncovered 386 mRNA Ψ sites (>10% Ψ fraction) shared among 2–3 human cell lines (Supplementary Fig. 3e). For highly modified Ψ (>50% Ψ fraction), we identified 127 cell-line-specific sites (Supplementary Fig. 3f) and 78 sites as highly modified Ψ in at least one human cell line and detectable (>10% Ψ fraction) in all three cell lines (Fig. 3e).

We next analyzed the motif frequency and modification fraction of all mRNA Ψ sites in all three cell lines. In HeLa cells, the most frequent motifs are GUΨCN (N = A or C or G or U), USΨAG (S = C or G), poly-U (UUUUU or more), NGΨGG (N = A or C or G or U) and GSΨGA (S = C or G) (Fig. 3f and Supplementary Fig. 3g). HEK293T and A549 cells also display the similar patterns in motif frequency (Supplementary Fig. 3h). Previously, GUΨC and UVΨAG (V = A or C or G) were reported as the potential TRUB1 motif and Pus7 motif, respectively^13^, which are consistent with our findings here. Note that we plotted the deletion ratio at each Ψ site versus the RPKM value of the corresponding mRNA (Supplementary Fig. 3i), which gives an estimated RPKM of 1.5 as the expression limit for mRNA Ψ detection under the current sequencing depth of ~80 M reads per library.

### Pseudouridine writers for Ψ deposition in HeLa mRNA

Thirteen putative PUS enzymes have been annotated in the human genome^7–9^, with dyskerin pseudouridine synthase 1 (DKC1) known to rely on H/ACA snoRNAs to guide pseudouridine deposition^25–27^. Most other PUS enzymes are thought to be stand-alone enzymes that function without snoRNAs^9,28^. To identify PUS enzymes that catalyze Ψ deposition at individual sites in mRNA, we performed shRNA knockdown of eight known PUS enzymes in HeLa cells followed by BID-seq. We noticed substantially reduced Ψ modification in shControl versus wild-type HeLa cells, most probably due to either cellular stress or immune stimulation caused by lentivirus transfection. We were still able to detect 133 mRNA Ψ sites with Ψ fractions above 10% in shControl HeLa cells and used these 133 sites to study Ψ deposition by writers under the same lentivirus infection conditions (Supplementary Table 8). We compared the deletion rate at each site among shControl and each PUS knockdown. For example, the highly modified Ψ site in *ERH* mRNA 3′-UTR displayed a Ψ fraction reduction from 96% to 8% upon TRUB1 knockdown, whereas knockdown of other PUS enzymes did not affect this site (Fig. 3g), revealing that this Ψ site is installed by TRUB1 (ref. ^13^). TRUB1 regulated 70 sites out of 133 (Fig. 3h,i), including 15 highly modified sites (>50% fraction) in transcripts such as *ERH*, *ZNF664*, *DKC1*, *M6PR*, *AGPAT5*, *SCP2*, *CDC6*, INTS1, FKBP4, *AMFR*, etc. (Supplementary Fig. 4a), out of which *ERH*, *ZNF664*, *DKC1*, *M6PR*, *AGPAT5*, *SCP2*, INTS1, FKBP4, *AMFR* and *HEXA* were also identified by Ψ-seq^5^. We then analyzed the motif frequency and modification fraction of the 70 TRUB1-regulated mRNA Ψ sites. The most frequent motifs are GUΨCN (N = A or C or G or U) and poly-U (UUUUU or more Us) (Supplementary Fig. 4b), consistent with the same main motif contexts revealed by BID-seq (Fig. 3f and Supplementary Fig. 3g).

PUS7 (refs. ^29–32^), PUS1 (refs. ^4,32^), PUS3, PUS7L, PUSL1, TRUB2 and DKC1 (refs. ^25–27^) also deposited 40, 28, 30, 24, 28, 28 and 33 Ψ sites in HeLa transcripts, respectively (Fig. 3j and Supplementary Fig. 4c). Overall, we found that 104 (out of 133) Ψ sites (in shControl HeLa cells) responded to knockdown of these eight PUS enzymes, with some sites regulated by one specific PUS enzyme and others affected by multiple PUS enzymes (Fig. 3k). The remaining 29 (out of 133) HeLa mRNA Ψ sites might be regulated by other PUS enzymes as PUS10 (ref. ^33^), RPUSD1, RPUSD2, RPUSD3 and RPUSD4 (ref. ^32^). Note that more effective knockdown or knockout with deeper sequencing may help confidently assign ‘writer’ proteins for all 133 mRNA Ψ sites in shControl cells.

### BID-seq detects abundant Ψ sites in mRNA from mouse tissues

**Fig. 4:**
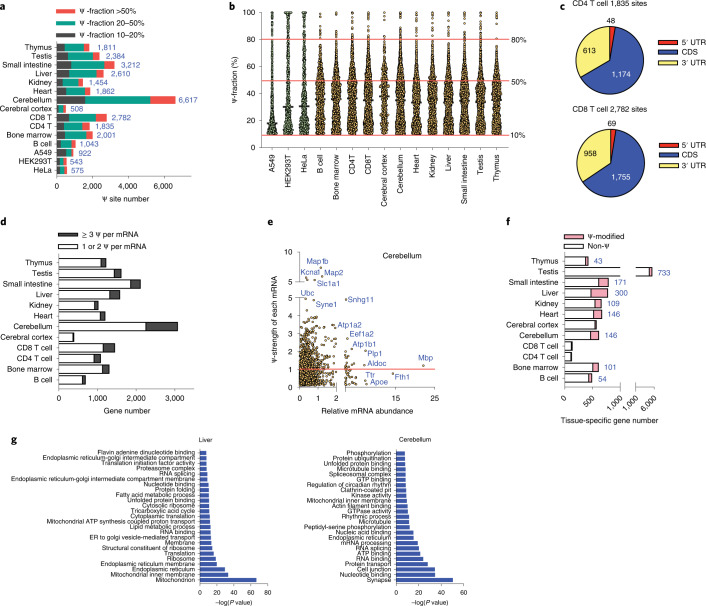
Mouse tissue mRNAs are heavily modified with Ψ. **a**, BID-seq reveals a large number of Ψ sites (modification fraction >10%) in 12 mouse tissues, with the Ψ site number in three human cell lines shown for comparison. **b**, Modification level distribution of mRNA Ψ sites in 12 mouse tissues, in which a number of Ψ sites are highly modified (modification fractions above 50%). The modification level distribution of Ψ sites in three human cell lines are shown as comparison. **c**, Pie chart showing the distribution of mRNA Ψ sites in CD4 T and CD8 T cells, with stoichiometry ≥10% in three mRNA segments. **d**, The number of Ψ-modified genes (with Ψ sites >10% fraction) that contain one or two Ψ versus above three Ψ sites per mRNA, in 12 mouse tissues. **e**, 2D plot of Ψ-modified genes (Ψ-fraction above 10% for each Ψ site) in mouse cerebellum, respectively, with ‘*x* axis’ as the mRNA abundance normalized to *Rps8* (abundant nontarget gene, without any Ψ on mRNA) and ‘*y* axis’ showing the Ψ-strength of each gene, defined as the sum of Ψ fraction at all the Ψ sites within one mRNA. The cutoff of Ψ-strength 1.0 was marked by a red line. **f**, Among tissue-specific genes in each tissue type, the gene number distribution of non-Ψ-modified genes versus Ψ-modified genes. **g**, Top 25 enriched GO clusters from nontissue-specific Ψ-modified genes, in mouse liver and cerebellum, respectively. One-sided Fisher’s exact test. Adjusted *P* values using the linear step-up method. Source data

To further investigate mRNA pseudouridylation in real tissues, we performed BID-seq with polyA-tailed RNA isolated from 12 mouse tissues. We detected many more Ψ candidate sites in mouse tissue mRNA than in HeLa mRNA, consistent with the trend shown in our LC-MS/MS measurements (Supplementary Fig. 5a) and a previous analysis of mouse brain and lung tissues^6^. Specifically, we identified 1,043, 2,001, 1,835, 2,782, 508, 6,617, 1,862, 1,454, 2,610, 3,212, 2,384 and 1,811 Ψ sites (>10% fraction) in mRNA from mouse B cell, bone marrow, CD4 T cell, CD8 T cell, cerebral cortex, cerebellum, heart, kidney, liver, small intestine, testis and thymus, respectively (Fig. 4a and Supplementary Tables 9–20). We observed a number of highly modified sites (>50% Ψ fraction) in all 12 tissues, particularly ranging from 50% to 80% fraction (Fig. 4b). Similar to human cell lines, mRNA Ψ in mouse tissues also accumulate in CDS and 3′ UTR (Fig. 4c). In metagene profiles, the mRNA Ψ sites in mouse liver, kidney, thymus and CD8 T cells, shown as examples, distribute in the CDS and 3′-UTR, with accumulation around the stop codon (Supplementary Fig. 5b).

In total we identified 4,008 highly modified mRNA Ψ sites (>50%) from all 12 tissues (Fig. 4a,b). We next asked whether some of these Ψ sites could be tissue specific and potentially distinguish tissue type. In all, 2,595 out of 4,008 Ψ sites were indeed tissue-specific and can serve as tissue-specific mRNA markers (Supplementary Fig. 5c,d). Particularly, we observed many tissue-specific Ψ sites in cerebellum, CD8 T cell, small intestine and testis mRNA. Whereas Ψ sites may serve as cell-type specific markers, highly modified Ψ sites are also shared among multiple tissues, suggesting common functions. Out of 4,008 sites, 462 display a Ψ fraction of over 50% in at least one tissue and are detectable (above 10% Ψ fraction) in at least four tissues (Supplementary Fig. 5e). It will be interesting to explore the functional roles of these shared mRNA Ψ sites in tissues in the future.

Another interesting observation is the presence of multiple Ψ sites (≥3 Ψ) per mRNA in a portion of pseudouridylated mRNAs in mouse tissues (Fig. 4d), especially in cerebellum, liver, CD4 T cells and CD8 T cells. For instance, around 25% of Ψ-modified genes in cerebellum carry at least three Ψ per mRNA. We used ‘Ψ-strength’ (defined as the sum of Ψ fraction in all Ψ sites in one gene) to measure and describe the overall level of Ψ modification in one gene. We then plotted Ψ-strength versus normalized gene expression level (normalized to the abundant housekeeping gene *Rps8*, which lacks detectable Ψ sites) to group all Ψ-modified genes, with Ψ-strength of 1.0 as the cutoff (Fig. 4e). We then investigated gene expression levels and found, compared with the genes of lower Ψ-strength (<1.0), those with high Ψ-strength (>1.0) displayed a notably higher expression level in tissues such as cerebellum, CD4 T cells, CD8 T cells, thymus and testis (Supplementary Fig. 6a), suggesting that Ψ deposition on mouse tissue mRNA might contribute to mRNA stability.

To further study the features of Ψ-modified genes, we grouped tissue-specific genes in each tissue type (defined as genes that show a much higher expression in one tissue versus all other tissues), and analyzed how many of them are Ψ-modified in each tissue. Notably, 16%, 24%, 22%, 16%, 38% and 22% of tissue-specific transcripts are Ψ-modified in bone marrow, cerebellum, heart, kidney, liver and small intestine (Fig. 4f). Collectively, our data suggest that pseudouridylation occurs in many tissue-specific mRNAs in mouse and may affect tissue-specific biological functions.

We next investigated the potential functions of nontissue-specific genes in each tissue type. GO analysis of these genes in each tissue type showed common functional clusters on endoplasmic reticulum, ribosome, ATP binding, nucleotide/RNA binding, etc., which display similarity to those in human cell lines (Fig. 4g and Supplementary Figs. 3d and 6b). Overall, mouse tissues clearly show abundant Ψ modifications in nucleus-encoded mRNA; some of these are shared across tissues, suggesting common functions.

In addition, we investigated Ψ modification on mitochondrion-encoded mRNAs and detected five Ψ sites in *ND1*, *CO1* and *ND4*, with Ψ stoichiometry at around 20–60%, from cultured human cell lines (Supplementary Fig. 6c). PUS1 seems to serve as the ‘writer’ protein for at least one Ψ site on *ND4* mRNA in HeLa cells (Supplementary Fig. 6d). However, Ψ is more abundant on mitochondrial mRNAs from diverse mouse tissues; we detected 66 mt-mRNA Ψ sites in multiple mt-mRNAs, with around 20–65% Ψ fraction (Supplementary Fig. 6e). In some tissues, several mt-mRNAs, such as *Nd1*, *Nd2*, *Nd4*, *Nd5*, *Co1* and *Atp6*, contain multiple Ψ modifications. Functional consequences of these mt-mRNA Ψ modifications require future investigations.

### Ψ increases mRNA stability

In mouse tissues, mRNAs with high Ψ strength tend to be more abundant (Supplementary Fig. 6a). Pseudouridylation of synthetic mRNA has been reported to increase its stability^34^; however, the extent and potential functions of pseudouridylation in native mRNA are poorly understood. As we show here that TRUB1 is a main enzyme that deposits Ψ on mRNA in HeLa cells, we investigated its potential role on transcript stability. Yeast *Pus4* (paralog of human TRUB1) overexpression is known to increase cell size and proliferation^35^. We also found consistently that TRUB1 depletion could inhibit cell growth, arrest cells in G1 phase, and cause reduced cell size (Supplementary Fig. 7a–d). We further validated the discovered Ψ sites in mRNA and also the TRUB1 function as a ‘writer’ protein using the CMC-treatment-based^4,5^ method for the four highly modified mRNA Ψ sites known to be installed by TRUB1, such as *ERH*, *SCP2*, *AMFR* and *CDC6* (Supplementary Fig. 7e,f); the CMC-based RT with quantitative PCR (RT-qPCR) assay worked well in single-site Ψ determination and displayed notably reduced readthrough ratio at Ψ sites on these four mRNAs, after CMC-treatment and normalization to control regions. We also verified an array of HeLa mRNA Ψ sites in different motif contexts using this orthogonal assay (Supplementary Fig. 7f,g). Furthermore, we employed the published ‘CMC-RT and ligation-assisted PCR analysis of Ψ modification’ (CLAP)^36^, for visualization and quantification of mRNA Ψ site by gel electrophoresis. We selected three Ψ sites with surrounding sequences suitable for the CLAP protocol and validated our BID-seq methods in both Ψ site detection and Ψ stoichiometry estimation (Supplementary Fig. 7h, i).

**Fig. 5:**
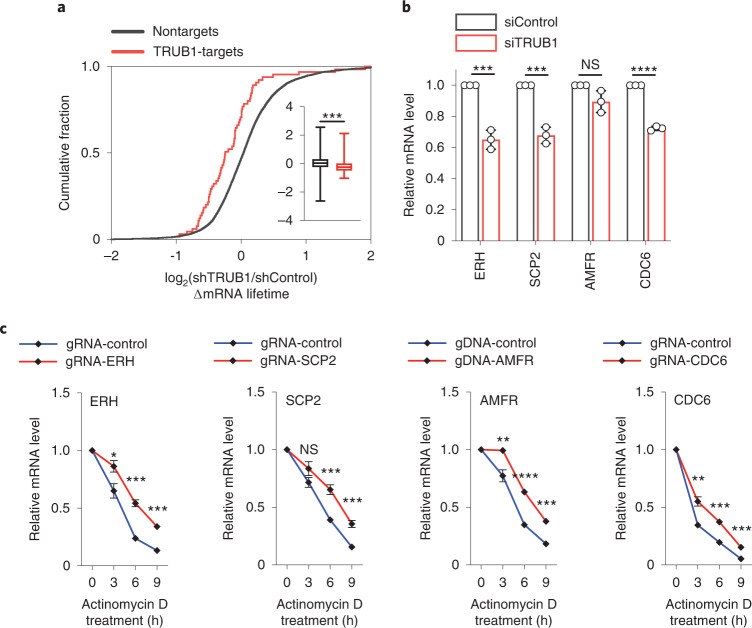
Ψ affects mRNA stability. **a**, Cumulative distribution showing the decreased mRNA half-life for TRUB1-targets in TRUB1-depleted HeLa cells versus the control, compared with nontargets. n = 7,881 nontargets, and n = 65 TRUB1-targets. Box, first and third quartiles; line in the middle of the box, median; short line, maximum and minimum; ***P = 0.0008; unpaired, two-tailed *t*-test. **b**, Relative mRNA levels of four representative transcripts carrying TRUB1-regulated highly modified Ψ, in siTRUB1 versus siControl. *P* = 0.0006, 0.0005, 0.0612 and <0.0001, respectively; unpaired, two-tailed *t*-test. **c**, Stable expression of dCas13d-TRUB1, by gRNA transfection, restored Ψ and increased half-life of the target mRNA in TRUB1-depeted HeLa cells. For ERH: *P* = 0.0104, 0.0002 and 0.0002, respectively; unpaired, two-tailed *t*-test. For SCP2: *P* = 0.0511, 0.0006 and 0.0006, respectively; unpaired, two-tailed *t*-test. For AMFR: *P* = 0.0025, <0.0001 and 0.0002, respectively; unpaired, two-tailed *t*-test. For CDC6: *P* = 0.0015, 0.0002 and 0.0002, respectively; unpaired, two-tailed *t*-test. For **b**–**c**, *n* = 3, biologically independent samples; data are presented as mean values ± s.d.; NS, *P* ≥ 0.05; *P < 0.05; **P < 0.01; ***P < 0.001 and ****P < 0.0001. Source data

We then performed TRUB1 knockdown and studied its effects on transcript half-life by RNA-seq. We noticed that TRUB1-targets, which carry TRUB1-modified Ψ in mRNA in shControl cells, displayed a shorter half-life upon TRUB1 knockdown, whereas the half-life of nontargets (without detectable Ψ) remained unchanged (Fig. 5a). We investigated the four representative genes containing TRUB1-regulated highly modified mRNA Ψ sites, *ERH*, *SCP2*, *AMFR* and *CDC6* (Supplementary Fig. 4a). Three of the four targets displayed notable reduced mRNA level after 72-h siTRUB1 knockdown compared with the control (Fig. 5b). By using RT-qPCR, we validated that TRUB1 depletion reduced the stability of all four representative TRUB1-targets but not a nontarget mRNA (Supplementary Fig. 7j), confirming that Ψ installed by TRUB1 stabilizes the target mRNA. To further validate the transcript stabilization role of TRUB1-regulated Ψ, we engineered a fused dCas13d-TRUB1 system^37^ and confirmed that site-specific Ψ deposition could notably prolong mRNA lifetime (Fig. 5c). Taken together, our data reveal a main functional role of pseudouridylation in stabilizing target mRNA.

### Pseudouridylation at mRNA stop codons

**Fig. 6:**
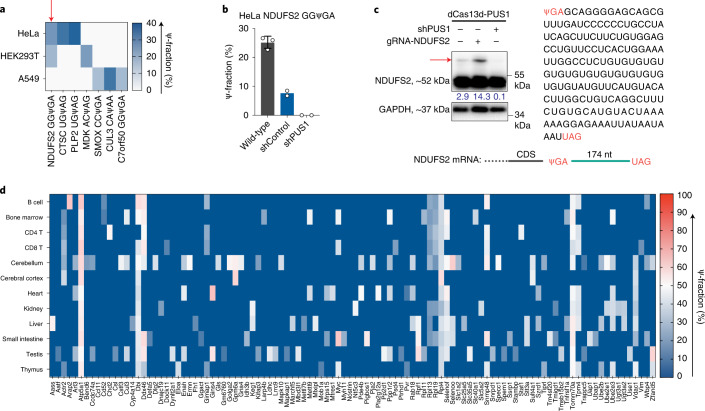
The presence of Ψ promotes stop codon readthrough in vivo. **a**, Heatmap plot of Ψ fraction for seven Ψ sites within mRNA stop codon in three human cell lines, in a matrix of the corresponding gene name versus each cell line. **b**, Ψ modification fraction of Ψ within stop codon of the *NDUFS2* mRNA, in wild-type HeLa cells, shControl HeLa cells and PUS1-depleted HeLa cells, respectively. For wild-type HeLa cells, *n* = 3, biologically independent samples; data are presented as mean values ± s.d. For shControl and PUS1-depleted HeLa cells, *n* = 2, biologically independent samples. **c**, Stop codon readthrough for the *NDUFS2* mRNA in HeLa cells investigated by immunoblotting assay. shControl or shPUS1 HeLa cells stably expressing dCas13d-PUS1 were transfected with control or *NDUFS2* gRNA for 48 h. The percentage numbers of readthrough ratio are shown in blue. The readthrough protein bands are labeled by red arrow. **d**, Heatmap plot of Ψ-fraction for 106 Ψ sites within mRNA stop codons in mouse tissues, in a matrix of the corresponding gene name versus tissue type. Source data

Using an in vitro translation assay, Karijolich et al. discovered a unique function that targeted pseudouridylation could convert nonsense codons into sense codons and promote readthrough (Supplementary Fig. 8a)^17^. More recently, it was demonstrated that Ψ can facilitate noncanonical base pairing in the ribosome decoding center to promote nonsense suppression^18,19^. Despite these important observations, whether Ψ naturally exists in stop codons of mRNA and promotes stop codon readthrough in vivo remains unclear. In HeLa, HEK293T and A549 cells, BID-seq revealed several pseudouridylation sites in stop codons (as ‘ΨGA’, ‘ΨAA’ and ‘ΨAG’) in *NDUFS2*, *CTSC*, *PLP2*, *MDK*, *SMOX*, *CUL3* and *C7orf50* mRNAs, with Ψ fraction ranging from 10% to 40% (Fig. 6a). The modification fraction of the ΨGA stop codon in *NDUFS2* mRNA decreased dramatically upon PUS1 knockdown (Fig. 6b). Correspondingly, we observed decreased stop codon readthrough for *NDUFS2* with PUS1 knockdown, whereas dCas13d-PUS1 coupled with guide RNA (gRNA) for *NDUFS2* substantially increased stop codon readthrough from around 3% up to ~14% (Fig. 6c and Supplementary Fig. 8b,c).

We also identified 106 Ψ-modified stop codons from 12 mouse tissues, with Ψ fraction ranging from 10% to 65% (Fig. 6d). In all cases, a nearby second stop codon without Ψ was found at downstream locations. Ψ-modified stop codons in *Atp5a1*, *Dbi*, *Rpl4* and *Tomm70a* are conserved in 11 or 12 tissues whereas others are tissue specific (Fig. 6d). Taken together, our data reveal the existence of Ψ in stop codons in native mRNAs, suggesting their role in promoting stop codon readthrough in vivo as an alternative translation regulation mechanism.

Among Ψ-modified stop codons from mouse tissues (Fig. 6d), we examined corresponding proteins that may include potential readthrough peptide with over 10% increased protein molecular weight (Supplementary Table 23) to allow for confident detection of the shifted protein band. We selected ten proteins with available commercial antibodies, and tested these proteins in seven different mouse tissues. Among these ten targets containing Ψ-modified stop codons, we observed notable band shifts for potential readthrough in *Selenof*, *Ppp1r2*, *Nt5c3*, *Szrd1* and *Cd52* (Supplementary Fig. 8d). These band shifts could be observed in four different mice repeats (Supplementary Fig. 8e) with some individual variations. The highest estimated readthrough is around 35% for *Selenof* in kidney with a stop codon modified by Ψ at around 42% stoichiometry (Supplementary Fig. 8d,e). Note that for *Selenof* and *Ube2e3*, there were no observable band shifts in some tissues, though BID-seq indicates the presence of Ψ-modified stop codon (Supplementary Fig. 8d). Interestingly, although BID-seq data reveals an approximately 12% Ψ-modified stop codon of *Cd52* mRNA from bone marrow but not any other tissues, we saw a strong band shift for *Cd52* readthrough peptide in bone marrow (Supplementary Fig. 8d,e), likely driven by a low-Ψ-modified stop codon. These observations suggest that the Ψ-mediated stop codon readthrough may depend on sequence context and is regulated by unknown tissue-specific mechanisms. A lot more future research is required to understand and potentially take advantage of this intriguing translation regulation mechanism.

## Discussion

Pseudouridine (Ψ) is one of the most abundant RNA modifications in mammalian mRNA. The 0.1–0.25% of Ψ/U ratio on polyA-tailed RNA measured by LC-MS/MS^6^ (Supplementary Fig. 5a) is comparable with the abundance of m^6^A in mammalian mRNA. Whereas mammalian mRNA m^6^A is installed mostly by one methyltransferase complex of METTL3-METTL14 (refs. ^38,39^), there are 13 annotated pseudouridine synthetases^7–9^ in the human genome that may install Ψ to different RNA species including mRNA. While studies of pseudouridylation of relatively abundant RNA species such as rRNA and tRNA have led to functional understandings, functional roles of Ψ in mRNA and other low abundant RNA species remain unclear, mostly because of the lack of a quantitative method that can not only map Ψ sites at the base resolution but also reveal the exact modification stoichiometry at individual sites.

To resolve this challenge, we report BID-seq for quantitative and comprehensive Ψ mapping in different species of RNA. This method can be applied to 10–20 ng input RNA, allowing RNAs isolated from precious samples to be analyzed. Instead of 5–30% formation of Ψ-BS in RBS-seq, we can induce close to stoichiometric formation of Ψ-BS with the new protocol. The BID-seq bisulfite reaction condition afford high deletion ratios on most motif contexts (Fig. 1e). This new method and the datasets available will greatly aid future functional investigations on Ψ.

The quantitative nature of BID-seq allowed us to reveal sequence motifs of pseudouridylation and assign pseudouridine synthase to individual sites in knockdown experiments in cell lines. Different from mammalian mRNA m^6^A methylation, in which a methyltransferase complex installs most methylation sites, pseudouridylation of mammalian mRNA seems to be more heterogeneous with multiple enzymes involved. While TRUB1 is responsible for a large portion of Ψ deposition in mRNA, other PUS enzymes we tested seem to contribute to mRNA pseudouridylation to various degrees, including DKC1, which uses snoRNA to guide pseudouridylation^25–27^.

While we uncovered hundreds of confident Ψ sites in multiple human cell lines, we identified a lot more frequently modified Ψ sites in mouse tissues. We consistently detected a few thousand Ψ sites with higher than 10% modification stoichiometry in most of these tissue mRNAs (Fig. 4a,b). For example, we observed 6,617 Ψ sites with stoichiometry higher than 10% in mouse cerebellum. Among all 12 tissues, the 4,008 highly modified Ψ sites (>50% fraction) in tissues include both tissue-specific and tissue-shared sites in numerous transcripts. These observations may suggest common as well as tissue-specific roles of mRNA pseudouridylation. Our new method and the datasets obtained offer critical resources for future functional exploration of RNA pseudouridylation in tissues and cell lines. Pseudouridylation may also represent new biomarkers to differentiate cell types in healthy as well as diseased samples^40,41^.

Our sequencing results suggested to us that mRNA pseudouridylation tends to correlate positively with transcript levels. Indeed, when we knocked down TRUB1, we observed a transcript stabilization effect of Ψ installed by TRUB1 (Fig. 5a,b). We tethered TRUB1 to dCas13d and demonstrated that Ψ installation by TRUB1 can stabilize the target mRNA (Fig. 5c). It is interesting to note that, among the two most abundant mammalian mRNA modifications, m^6^A and Ψ, one tends to destabilize mRNA^42,43^, and the other tends to stabilize mRNA. Finally, we reveal the presence of numerous Ψ sites at mRNA stop codons; some of these Ψ-modified stop codons could induce stop codon readthrough in specific tissues (Supplementary Fig. 8). We show that pseudouridylation writer tethered with dCas13d can be guided by designed gRNA to install Ψ at the stop codon for readthrough, offering a potential strategy to overcome human diseases associated with premature stop codons in specific tissues^44,45^.

### Limitations of the study

The detection of Ψ sites by BID-seq is based on the deletion signature generated at Ψ-BS sites during the RT process. Sufficient reads coverage might be required to detect low-modified Ψ sites on low-expressed RNAs. When the Ψ site is neighbor to one or more uridines, it is difficult to determine the exact pseudouridylation site because the same deletion pattern would be generated with Ψ at any site. When this is the case, we can use CMC-based RT stop to validate which uracil is truly pseudouridylated. BID-seq generates Ψ-BS adduct with high efficiency and induces more than 50% deletion rates at the Ψ site in 232 of 256 motif contexts (NNΨNN), with very low background deletion rate (<1%) at untreated Ψ sites (Fig. 1c–e). The unmodified probes containing 0% Ψ (NNUNN) do show background deletion (around 10–25% deletion ratio) in a few motifs after BID-seq treatment (Supplementary Fig. 1c), we eliminate these potential false positives using the current analysis pipeline with stringent criteria to call Ψ candidate sites.

## Methods

### Cell culture

HeLa, HEK293T and A549 cells were purchased from the American Type Culture Collection (ATCC). 293TN cells were purchased from System Bioscience. Cells were cultured at 37 °C with 5.0% CO_2_ in a Heracell VIOS 160i incubator (Thermo Scientific). All cell lines were grown in DMEM medium (GIBCO, catalog no. 11995) supplemented with 10% v/v FBS and 1% penicillin/streptomycin (Gibco). The percentage of surviving cells after treatment was assessed by the SRB assay^46^. Cell cycle distribution and cell size determination were assessed by flow cytometry.

### Antibodies

The following antibodies were used in this study: rabbit monoclonal anti-NDUFS2, clone EPR16266 (abcam, catalog no. ab192022, 1:1,000), mouse monoclonal anti-GAPDH, clone 0411 (Santa Cruz, catalog no. sc-47724, 1:1,000), rabbit polyclonal anti-SELENOF (My BioSource, catalog no. MBS3208942, 1:500), mouse monoclonal anti-UBE2E3, clone OTI7E8 (Novus Biologicals, catalog no. NBP2-03819, 1:500), rabbit polyclonal anti-PPP1R2 (Thermo Fisher Scientific, catalog no. PA5-115787, 1:500), rabbit polyclonal anti-NT5C3 (Proteintech, catalog no. 11393-1-AP, 1:500), rabbit polyclonal anti-SZRD1 (Thermo Fisher Scientific, catalog no. A304-742A, 1:1,000), rabbit polyclonal anti-SNRPD1 (Novus Biologicals, catalog no. NBP2-36427, 1:500), rabbit polyclonal anti-DNAJC19 (Thermo Fisher Scientific, catalog no. PA5-98770, 1:1,000), rabbit polyclonal anti-MAPKAP1 (Proteintech, catalog no. 15463-1-AP, 1:500), rabbit monoclonal anti-CD52, clone EPR3153(2) (abcam, catalog no. ab125071, 1:1,000), rabbit polyclonal anti-A2LD1 (Proteintech, catalog no. 23280-1-AP, 1:500), anti-rabbit IgG, horseradish peroxidase (HRP)-linked antibody (Cell Signaling, catalog no. 7074S WB, 1:2,000), anti-mouse IgG, HRP-linked antibody (Cell Signaling catalog no. 7076S, WB 1:2,000).

### shRNA knockdown and plasmid transfection

For transient transfection, cells were transfected with casRx (Addgene catalog no. 109053) gRNA construct and pCMV-TRUB1-dCas13D by Lipofectamine 2000 Transfection Reagent (Invitrogen) according to manufacturer’s protocol, or with siRNA by Lipofectamine RNAiMAX Transfection Reagent (Invitrogen) following commercial protocols. For lentivirus production, a lentiviral construct (pLKO-Tet-On for inducible knockdown of pseudouridine synthetases, or green fluorescent protein as a negative control) together with pMD2.G (Addgene catalog no. 12259) and psPAX2 (Addgene catalog no. 12260) were cotransfected into 293TN cells (System Biosciences) as previously described^47^. Viruses were concentrated by the PEG-it Virus Precipitation Solution and used for infecting cells in the presence of TransDux (System Biosciences). Transfected cells were selected by 2 μg ml^–1^ puromycin. To generate the dcas13D-TRUB1 or PUS1 cells, HeLa cells were transfected with PB-Cuo–TRUB1 (or PUS1) -dCas13D-IRES-GFP-EF1α-CymR-Puro construct following commercial protocol (System Biosciences). Pools of stable transfectants were selected by antibiotics or sorted by flow cytometry. Doxycycline (1 μg ml^–1^) was used to induce shRNA while cumate (30 μg ml^–1^) was used to induce gRNA expression. The shRNA, siRNA and gRNA sequences are listed in Supplementary Table 21.

### RNA isolation

Total RNA isolation: (1) mouse tissues were weighted and homogenized in TRIzol reagent (Invitrogen) until no visible chunks were left, while cultured cells could be smoothly suspended in TRIzol reagent; (2) cellular total RNA was isolated according to the TRIzol reagent manufacturer’s protocol, followed by isopropanol precipitation; (3) when we extracted rRNA-depleted total RNA for a typical RNA-seq or lifetime sequencing, RiboMinus Eukaryote System v.2 (Invitrogen) was further used for rRNA removal;

polyA-tailed RNA isolation: Dynabeads mRNA DIRECT Purification Kit (Invitrogen) was used for polyA^+^ RNA enrichment.

### BID-seq for Ψ site detection

Wild-type HeLa, HEK293T or A549 cells (three replicates for each sample, one 3.5-cm plate per replicate); HeLa shControl or PUS knockdown cells were prepared as described (two replicates for each sample, one 10-cm plate per replicate); mouse tissues, except immune cells, were collected from two replicates (one wild-type mouse per replicate), age- and sex-matched. To harvest enough material, mouse immune cells such as B cells, CD4 T cells and CD8 T cells were collected and combined from two replicates (eight wild-type mice per replicate), again age- and sex-matched. After extracting the polyA^+^ RNA from HeLa cells or mouse tissues, around 10–20 ng RNA was fragmented using RNA Fragmentation Reagents (catalog no. AM8740, Invitrogen) and heated at 70 °C for 14 min, followed by purification with RNA Clean and Concentrator (Zymo Research). 3′-End repair and 5′-phosphorylation were conducted with T4 polynucleotide kinase (PNK) (catalog no. EK0032, Thermo Fisher Scientific). RNA was mixed with 3 µL 10× T4 PNK reaction buffer (catalog no. B0201S, NEB) and 3 µl T4 PNK, diluted to a final volume of 30 µl, and incubated at 37 °C for 45 min; then 1.5 µl T4 PNK and 1.5 µl 10 mM ATP were added for another incubation at 37 °C for 45 min, followed by RNA Clean and Concentrator (Zymo Research) purification eluting with 10 µl RNase-free water. To perform 3′-adapter ligation, 10 µl 3′-repaired and 5′-phosphorylated RNA fragments were incubated with 1.0 µl 20 µM RNA 3′ SR Adapter (5′App-NNNNNATCACG AGATCGGAAGAGCACACGTCT-3SpC3, with ATCACG as the inline barcode) at 70 °C for 2 mins and placed immediately on ice. Then, 2.5 µl 10× T4 RNA Ligase Reaction Buffer (catalog no. M0373L, NEB), 7.5 µl PEG8000 (50%), 1 µl SUPERase•In RNase Inhibitor and 2 µl T4 RNA Ligase 2 truncated KQ (catalog no. M0373L, NEB) were added to the RNA–adapter mixture^48,49^. The reaction was incubated at 25 °C for 2 h followed by 16 °C for 10 h. The reaction was further diluted to 47 µl with nuclease-free water, and the excessive adapters were removed with 2 µl 5′-deadenylase (catalog no. M0331S, NEB) at 30 °C for 30 min followed by adding 1 µl RecJf (catalog no. M0264L, NEB) for ssDNA digestion at 37 °C for 30 min. The 3′-end-ligated RNA was extracted by RNA Clean and Concentrator (Zymo Research) and eluted with 9.3 µl RNase-free water.

The purified RNA was incubated with 1.2 µl 10 µM 5′ SR Adapter (5′-GUUCAGAGUUCUACAGUCCGACGAUC NNNNN-3′) at 70 °C for 2 mins and placed immediately on ice. Then 2.5 µl 10× T4 RNA ligase reaction buffer, 1.0 µl 25 mM ATP, 10 µl PEG8000 (50%) and 1 µl T4 RNA Ligase 1 (high concentration, catalog no. M0437M, NEB) were added to the RNA–adapter mixture. The reaction was mixed well and incubated at 25 °C for 8 h, followed by RNA Clean and Concentrator (Zymo Research) purification, eluting with 10 µl RNase-free water.

A 1.5 µl aliquot of the purified RNA was saved for ‘Input’ library construction, 8.5 µl was subjected to BID-seq optimized bisulfite treatment, as the ‘Treated’ sample. The 8.5 µl RNA was mixed with 45 µl freshly prepared BID-seq BS reagent (2.4 M Na_2_SO_3_ and 0.36 M NaHSO_3_, prepared by dissolving 270 mg Na_2_SO_3_ and 34 mg NaHSO_3_ in 900 µl RNase-free water) and incubated at 70 °C for 3 h. Then, 75 µl RNase-free H_2_O, 270 µl RNA binding buffer (RNA Clean and Concentrator), and 400 µl ethanol were added to the reaction mixture, which was mixed well and loaded on a RNA Clean and Concentrator-5 column. After spinning and washing once with 200 µl RNA wash buffer (RNA Clean and Concentrator), 200 µl RNA Desulphonation Buffer (catalog no. R5001-3-40, Zymo Research) was added to the column and incubated at room temperature for 1 h. This was followed by spinning and washing twice with 700 µl RNA wash buffer (RNA Clean and Concentrator), followed by eluting RNA with 10.5 µl RNase-free water. Then the ‘Input’ was diluted to 10.5 µl with RNase-free water. Both ‘Input’ and ‘Treated’ samples were mixed with 1.0 µl 2.0 µM SR RT primer (5′-AGACGTGTGCTCTTCCGATCT -3′) at 65 °C for 2 min and moved immediately onto ice. To this was added 4 µl 5× SSIV Buffer, 2 µl 10 mM dNTP Solution Mix (NEB), 1 µl 100 mM dithiothreitol, 0.5 µl RNaseOUT Recombinant Ribonuclease Inhibitor (catalog no. 10777019, Thermo Scientific) and 1 µl SuperScript IV Reverse Transcriptase (SSIV, catalog no. 18090050, Thermo Scientific). The reaction was mixed well and incubated at 50 °C for 1 h, followed by adding 1 µl RNase H (catalog no. M0297L, NEB) and incubating at 37 °C for 20 min. The reaction mixture was heated at 70 °C for 5 min and then cDNA was purified using DNA Clean and Concentrator (Zymo Research). The eluted cDNAs (20 µl) were stored at –80 °C; 4 µl was used for each 15-cycle PCR amplification reaction, which was performed with the SR Primer for Illumina (NEB) and indexed primers (from NEBNext Multiplex Oligos for Illumina). All libraries were purified on a 3.5% low melting point agarose gel and sequenced on Illumina Nova-seq 6000 with single-end 100 bp read length.

### Reaction of model RNA oligonucleotides with BS and MALDI-TOF MS

To 9 μl of optimized BS reagent, 1 μl of synthetic RNA oligo AGXGA (X = C, U or Ψ, 100 ng μl^–1^) was added, followed by mixing well via pipetting. The reaction mixture was incubated in PCR instrument at 70 °C for 4 h. After cooling to room temperature, 10 μl Tris-HCl buffer (1.5 M, pH 8.8) was added and mixed well by pipetting. The mixture was incubated at 37 °C for 1 h for desulfonation. Then, 2 μl of the mixture was added to 40 μl resin (Bio-Rad) and allowed to stand at room temperature for 30 min. Then 1.8 μl supernatant was mixed with matrix 2′,4′,6′-trihydroxyacetophenone monohydrate and loaded onto a MALDI plate. The MALDI-TOF MS recorded the signals using negative reflector mode.

### LC-MS/MS

To measure Ψ in mRNA in HeLa and mouse tissues, mRNA was purified from total RNA by two rounds of polyA^+^ RNA enrichment and one round of rRNA removal as described in RNA isolation; 200 ng mRNA was dissolved in 11 μl water, and then mixed with 2.5 μl 100 mM NH_4_OAc (pH 5.2), 1 μl nuclease P1 (1U μl–^1^, Sigma-Aldrich) and 10.5 μl water, followed by an incubation at 42 °C for 3 h. Then, 3 μl freshly prepared 1.0 M NH_4_HCO_3_, 1 μl Fast AP (1 U μl^–1^, Invitrogen) and 1 μl water were added, and the reaction mixture was incubated at 37 °C for 3 h. Upon the completion of incubation, the reaction mixture was diluted to 50 μl and the samples filtered through 0.22 μm Millex-GV polyvinylidenedifluoride filters (Millipore). A 5 μl sample was then injected into a ZORBAX SB-Aq 4.6 × 50 mm column (Agilent) on UHPLC (Agilent) coupled to a SCIEX 6500+ Triple Quadrupole Mass Spectrometer in positive electrospray ionization mode. The nucleosides were quantified based on the nucleoside to base transitions: 268 to 136 (A), 282.1 to 150.1 (m^6^A), 245 to 113.1 (U), 245.1 to 125 (Ψ) and compared with calibration curves. Three biological independent replicates were used for Ψ level quantification, and each sample was injected three times.

### RNA-seq

Tet-On-shRNA cells were incubated with doxycycline with a final concentration of 1 μg ml^–1^ for 6 days or transient knockdown cells for 3 days before harvest. For RNA lifetime study, cells were treated with actinomycin D with a final concentration of 5 μg ml^–1^ for 0, 3, 6 and 9 h. Cells were then harvested by trypsinization, and total RNA was isolated as described above. ERCC ExFold RNA Spike-In Mix (Invitrogen) was added to 2 μg total RNA and subjected to two rounds of RiboMinus. For comparing RNA expression responsive to TRUB1 knockdown, mRNA was isolated as described above. Three biological independent replicates per condition were sequenced; 10 ng rRNA-depleted RNA was used to construct libraries with SMARTer Stranded Total RNA-Set Kit v.2 (Takara Bio USA).

### RT-qPCR

RT was performed using PrimeScript RT Reagents Kit (Takara Bio USA, catalog no. RR037A), according to the vendor’s protocol. A tenfold dilution of cDNA was used to measure relative transcript abundance by real-time PCR. FastStart Essential DNA Green Master (Roche) and QuantStudio 6 Pro Real-Time PCR System (Thermo Fisher Scientific) were used to conduct quantitative PCR. For each sample, technical triplicates were performed and normalized to the expression level of 18S rRNA and other internal standard genes. For mRNA lifetime study, 18S rRNA was used as internal control as its level is not affected by Actinomycin D treatment. To determine relative expression, the 2^−ΔCt^ method was used. Primers are listed in Supplementary Table 21.

### Immunoprecipitation and immunoblotting

Immunoprecipitation (IP) and immunoblotting (IB) were performed as previously described^50^. In brief, protein samples were isolated by RIPA buffer (1% Triton X-100, 150 mM NaCl, 20 mM Na_2_HPO_4_, pH 7.4) containing Halt Protease and Phosphatase Inhibitor Cocktail (Thermo Scientific). BCA assay (Thermo Scientific) was used to determine the protein concentration. For IP, the antibody was conjugated to protein A/G magnetic beads by incubation at 4 °C for 2 h, followed by washing three times and incubating with cell lysates at 4 °C overnight. Equal amounts of purified protein were separated by SDS–PAGE followed by wet transfer to polyvinylidenedifluoride membranes. Blots were blocked with 5% nonfat milk or BSA and incubated with the primary antibody at 4 °C overnight. Signals were detected by HRP-linked secondary antibodies (Cell Signaling) together with SuperSignal West Pico Plus chemiluminescent substrate (Thermo Scientific) and imaged in a FluorChem R system (ProteinSimple). For the stop codon readthrough detection, the bands of interest were excised and submitted to MS bioworks using the MSB03 service.

### Validation in Ψ sites assessed by CMC-assisted RT-qPCR assay

PolyA^+^ RNA from HeLa cells was subjected to CMC-treatment and purified by ethanol precipitation. For each highly modified Ψ site in HeLa mRNA, two types of RT-qPCR primers were designed for (1) Ψ-region: the 250-nucleotide (nt) region centered by the target Ψ site; (2) control-region: the 250-nt region within this mRNA, without overlapping with the Ψ-containing 250-nt region. The same amount of untreated or CMC-treated^4,5^ HeLa RNA was used for two separate RT reactions, with the RT primer for the Ψ-region and control-region, respectively. For each RT reaction, polyA^+^ RNA was incubated with each gene-specific RT primer at 70 °C for 2 min and then moved quickly onto ice. Then 2 µl 5× first strand buffer, 0.5 µl dithiothreitol (100 mM), 1 µl dNTP (10 mM), 0.5 µl RNaseOut and 1 µl SuperScript III reverse transcriptase were added to the RNA-primer mixture and diluted to a final volume of 20 µl. FastStart Essential DNA Green Master (Roche) and QuantStudio 6 Pro Real-Time PCR System (Thermo Fisher Scientific) were used for RT-qPCR quantitation. The readthrough ratio was calculated by the Cq values on Ψ-region normalized to the control-region, in CMC-treated sample versus untreated samples. Primer sequences are listed in Supplementary Table 22.

### Optimized CMC-RT and ligation-assisted PCR analysis of Ψ modification

CMC-RT and ligation-assisted PCR analysis of Ψ modification (CLAP) was performed as described previously^36^ with some modifications. HeLa polyA^+^ RNA was extracted from total RNA with Dynabeads mRNA DIRECT Purification Kit (Invitrogen). To 5.5 μg of polyA^+^ RNA (-CMC) in 12 μl RNase-free water, 28 μl 1× TEU Buffer (50 mM Tris-HCl (pH 8.3), 4 mM EDTA, 7 M urea) and 2 μl SUPERase•In RNase Inhibitor (20 U μl^–1^, Invitrogen, catalog no. AM2696) were added. To 8.3 μg polyA^+^ RNA (+CMC) in 12 μl RNase-free water, 24 μl 1× TEU Buffer, 4 μl 1.0 M CMC in 1× TEU Buffer and 2 μl SUPERase•In were added and incubated at 30 °C for 16 h. Next, 140 μl RNase-free water, 20 μl 3.0 M NaOAc (pH 5.2), 550 μl 100% ethanol and 1 μl GlycoBlue coprecipitant (15 mg ml^–1^, Thermo Scientific) were added, and RNA was precipitated overnight at –80 °C.

Next, samples were centrifuged at 7,000*g* at 4 °C for 30 min, the supernatant was removed and 1 ml 70% ethanol was added and incubated at –80 °C for 2 h. After that, samples were centrifuged again, supernatant was removed and dried pellet was resuspended in 40 μl 50 mM Na_2_CO_3_, 2 mM EDTA (pH 10.4) and 1 μl SUPERase•In and incubated at 37 °C for 6 h. Next, samples were purified by RNA Clean and Concentrator (Zymo Research) as follows: 260 μl RNA binding buffer and 300 μl 100% ethanol were added to each sample and, subsequently, the vendor’s protocol was followed. Recovered RNA underwent 5′ phosphorylation as follows: to 1 μg of CMC ± polyA^+^ RNA in 20 μl RNase-free water were added 2.5 μl SUPERase•In, 5 μl 10× T4 PNK Buffer, 2.5 μl 10 mM ATP and 5 μl T4 PNK (NEB; catalog no. M0201S). The volume was adjusted to 50 μl with RNase-free water and the reaction run at 37 °C for 1 h followed by purification with RNA Clean and Concentrator (Zymo Research).

All recovered polyA^+^ RNA (10 μl) was mixed with 1 μl 100 μM RNA-5 Blocking Oligo (/5AmMC6/rArCrCrCrA) and denatured at 65 °C for 2 min, followed by moving onto ice immediately. Then 3 μl 10× T4 RNA ligase buffer, 3 μl 10 mM ATP, 10 μl 50% PEG8000, 1 μl RNaseOUT recombinant ribonuclease inhibitor and 1 μl T4 RNA ligase I (NEB, catalog no. M0437M) were added, mixed well and incubated at 25 °C for 2 h then at 16 °C for 12 h. After that, samples were purified by RNA Clean and Concentrator (Zymo Research) as follows: 270 μl binding buffer and 300 μl 100% ethanol were added to each sample and the vendor’s protocol was then followed. Samples of 40 ng (–CMC) and 60 ng (+CMC) ligated polyA^+^ RNA underwent reverse transcription as follows: 2 μl (–CMC) and 3 μl (+CMC) ligation mixtures were mixed with 1 μl 1.0 μM target-specific primer and incubated at 65 °C for 2 min then put on ice. Then, 2 μl 10× AMV RT Buffer, 2 μl 10 U μl^–1^ AMR RT (NEB; catalog no. M0277L), 2 μl 10 mM dNTP, 0.5 μl Murine RNase inhibitor and the final volume was adjusted with water to 20 μl. RT was run for 1 h at 42 °C followed by denaturation at 80 °C for 5 min. Next, 1 μl RNase H (NEB, catalog no. M0297L) was added and the mixture incubated at 37 °C for 20 min followed by denaturation at 80 °C for 5 min. To 10 μl RT reaction mixture, 1.5 μl adapter/split oligonucleotide mixture (1.0 μM adapter; 1.5 μM splint) was added and incubated at 75 °C for 3 min, followed by moving onto ice. Then, 4 μl 10× T4 DNA ligase buffer, 5 μl DMSO, 1 μl 40 U μl^–1^ T4 DNA Ligase (NEB, catalog no. M0202L) and 18.5 μl H_2_O and incubated at 16 °C for 16 h followed by denaturation at 65 °C for 10 min. For PCR, 5 μl of the ligation mixture was mixed with 8 μl 5× Q5 reaction buffer, 0.8 μl 10 mM dNTPs, 2.4 μl of the mixture gene-specific forward/reverse primers (5 μM) and 0.5 μl Q5 high-fidelity DNA polymerase (NEB, catalog no. M0491L), followed by adjusting the volume to 40 μl. PCR product was amplified for 35 cycles at the following annealing temperatures: 70 °C for ERH, 64 °C for CDC6 and 65 °C for TRIP6. Then, 10 μl PCR mixture was mixed with 2 μl 6× TriTrack DNA Loading Dye (Thermo Fisher, catalog no. R1161). Samples and Low Range DNA Ladder (Thermo Fisher, catalog no. SM1193) were loaded at 4 °C onto pre-run 4–20% Novex TBE Gel (Invitrogen) and run at 10 V cm^–1^ at 4 °C. Gels were stained with SYBER gold nucleic acid gel stain (Thermo Fisher, catalog no. S11494) and imaged with a Bio-Rad Imaging System. Band intensity was quantified in Image Lab v.5.0 (Bio-Rad).

For 18S Ψ822 as a positive control, total RNA was treated as described above. PCR product was amplified for 25 cycles at the annealing temperature of 65 °C. All primer sequences are listed in Supplementary Table 22.

### Ψ modification fraction estimation

The 30-mer RNA probes with –NNΨNN– were used as the ‘100% Ψ’ standard. RNA oligonucleotides containing –NNUNN– were used as ‘0% Ψ’. The ‘100% Ψ’ and ‘0% Ψ’ standards were combined to generate six oligonucleotide mixtures at different methylation levels (100% Ψ, 80% Ψ, 60% Ψ, 40% Ψ, 20% Ψ, 0% Ψ). All oligonucleotide mixtures were subjected to BID-seq in parallel, the deletion rate patterns of each sequence context were analyzed and a fitting curve was plotted based on the relationship of observed deletion rate and Ψ fraction. The observed deletion rate *y* and Ψ fraction *x* can be expressed by the following equation:

$$y = \frac{{B + \left( {R - A \cdot R - B} \right) \cdot x}}{{1 - A \cdot x}}$$

where *A* is the dropout ratio of modified fragments, *B* is the background deletion rate (the deletion rate at unmodified U), and *R* is BID-seq induced deletion ratio. The parameters of *A*, *B* and *R* for each sequence context are provided in Supplementary Table 1.

### Sequencing data processing and analysis

The sequencing data were all trimmed with cutadapt tool to remove adapters and low-quality reads. PCR duplicates were removed with BBMap tool (v.38.73), 5-mer random barcodes at reads ends were trimmed and low-quality or short reads (less than 20 nt) were removed using cutadapt tool (v.1.15). Remaining reads were aligned to hg38 or mm10 genome using Tophat2 (v.2.1.1) and bowtie2 (v.2.4.0) allowing, at most, three mismatches. The generated.bam files were split into positive and negative strands and sorted using Samtools (v.1.9). Sequence variants were identified by measuring the base composition at each position using bam-readcount software (v.0.8.0). The generated bam-readcount results were parsed and analyzed by inhouse scripts. Internal deletion ratio at each Ψ candidate site suggested by Tophat2, was further calculated by data output from bam-readcount pipeline and confirmed by direct IGV visualization (v.2.8.0). In summary, one Ψ candidate site needs to satisfy the following criteria in its deletion profile: (1) deletion rate above 5% (with deletion count above five) in BID-seq libraries; (2) deletion rate below 1% in ‘Input’ libraries; (3) total reads coverage depth above 20 in both BID-seq and ‘Input’ libraries; (4) deletion rate above 1.5-fold over background in any given sequence motif (defined as the deletion rates detected from RNA probes containing 0% Ψ, as in Supplementary Fig. 1c); (5) we excluded uridine sites at the neighboring nucleotide 3′ or 5′ to known Ψ sites; (6) all deletion signatures must be from ‘U’ sites marked by hg38 or mm10 FASTA file, instead of from A or C or G. Under the current sequencing depth (around 80 M reads per library), we set RPKM = 1.5 as the lowest expression level for mRNA Ψ detection.

The ‘input’ samples of BID-seq, for both human cell lines and mouse tissues, are equivalent to regular RNA-seq; therefore, we quantified the gene-level read counts of input samples that aligned to hg38 or mm10 for gene expression analysis with Cufflinks software (v.2.2.1). GO analysis was performed using the online analysis software DAVID 2021 (https://david.ncifcrf.gov).

### Animal culture

C57BL/6J mice were purchased originally from the Jackson Laboratory (Strain no. 000664); 7-week-old male and female mice were used. Mice were housed in a virus-free facility at 21 ± 1 °C with a controlled 12-h light cycle (individually ventilated caging system (GM500)). The animals had access to standard chow and water ad libitum. The relative humidity was controlled at 55% ± 10%. All mouse experiments were approved by the University of Chicago Institutional Animal Care and Use Committee.

### Statistics and reproducibility

For BID-seq libraries, two or three biologically independent replicates were used in each experiment with cultured cells. Immunoblots are representative images from at least three rounds of independent experiments. Data are presented as the mean ± s.d., with two-tailed Student’s *t*-tests on the statistical significance of differences between groups. All statistical analysis and data graphing were done in Prism (v.9.2.0) software.

No statistical methods were applied to pre-evaluate sample size. No data were excluded from analysis. Samples in this study were not randomized. Blinding was not used for this study because cell culture, sample preparation, reagents and experimental settings were kept consistent for each experiment.

### Reporting summary

Further information on research design is available in the Nature Research Reporting Summary linked to this article.

## Online content

Any methods, additional references, Nature Research reporting summaries, source data, extended data, supplementary information, acknowledgements, peer review information; details of author contributions and competing interests; and statements of data and code availability are available at 10.1038/s41587-022-01505-w.

## Supplementary information

Supplementary Information Supplementary Figs. 1–8.

Reporting Summary

Supplementary Table 1 Supplementary Tables 1–23.

Supplementary Data 1 Unprocessed gels for Supplementary Fig. 7h.

Supplementary Data 2 Unprocessed blots for Supplementary Fig. 8d,e.

Supplementary Data 3 Statistical source data for Supplementary Fig.1.

Supplementary Data 4 Statistical source data for Supplementary Fig.2.

Supplementary Data 5 Statistical source data for Supplementary Fig.3.

Supplementary Data 6 Statistical source data for Supplementary Fig.4.

Supplementary Data 7 Statistical source data for Supplementary Fig.5.

Supplementary Data 8 Statistical source data for Supplementary Fig.6.

Supplementary Data 9 Statistical source data for Supplementary Fig.7.

Supplementary Data 10 Statistical source data for Supplementary Fig.8.

## Data Availability

Sequencing data are available in the Gene Expression Omnibus database under the accession number of GSE179798 (ref. ^51^). Source data are provided with this paper.

## Source data

Source Data Fig. 1 Statistical source data for Fig. 1.
Source Data Fig. 2 Statistical source data for Fig. 2.
Source Data Fig. 3 Statistical source data for Fig. 3.
Source Data Fig. 4 Statistical source data for Fig. 4.
Source Data Fig. 5 Statistical source data for Fig. 5.
Source Data Fig. 6 Statistical source data for Fig. 6.
Source Data Fig. 6 Unprocessed blots for Fig. 6c.
