# Supplementary material for: Quantitative sequencing using BID-seq uncovers abundant pseudouridines in mammalian mRNA at base resolution
Source: Nat Biotechnol. 2022 Oct 27;41(3):344–54. doi: 10.1038/s41587-022-01505-w (PMC10017504; doi:10.1038/s41587-022-01505-w)
Supplement: Supplementary file 1 — Supplementary Figs. 1–8. [file 41587_2022_1505_MOESM1_ESM.pdf]

nature biotechnology

Article

https://doi.org/10.1038/s41587-022-01505-w

Quantitative sequencing using BID-seq uncovers abundant pseudouridines in mammalian mRNA at base resolution

In the format provided by the authors and unedited

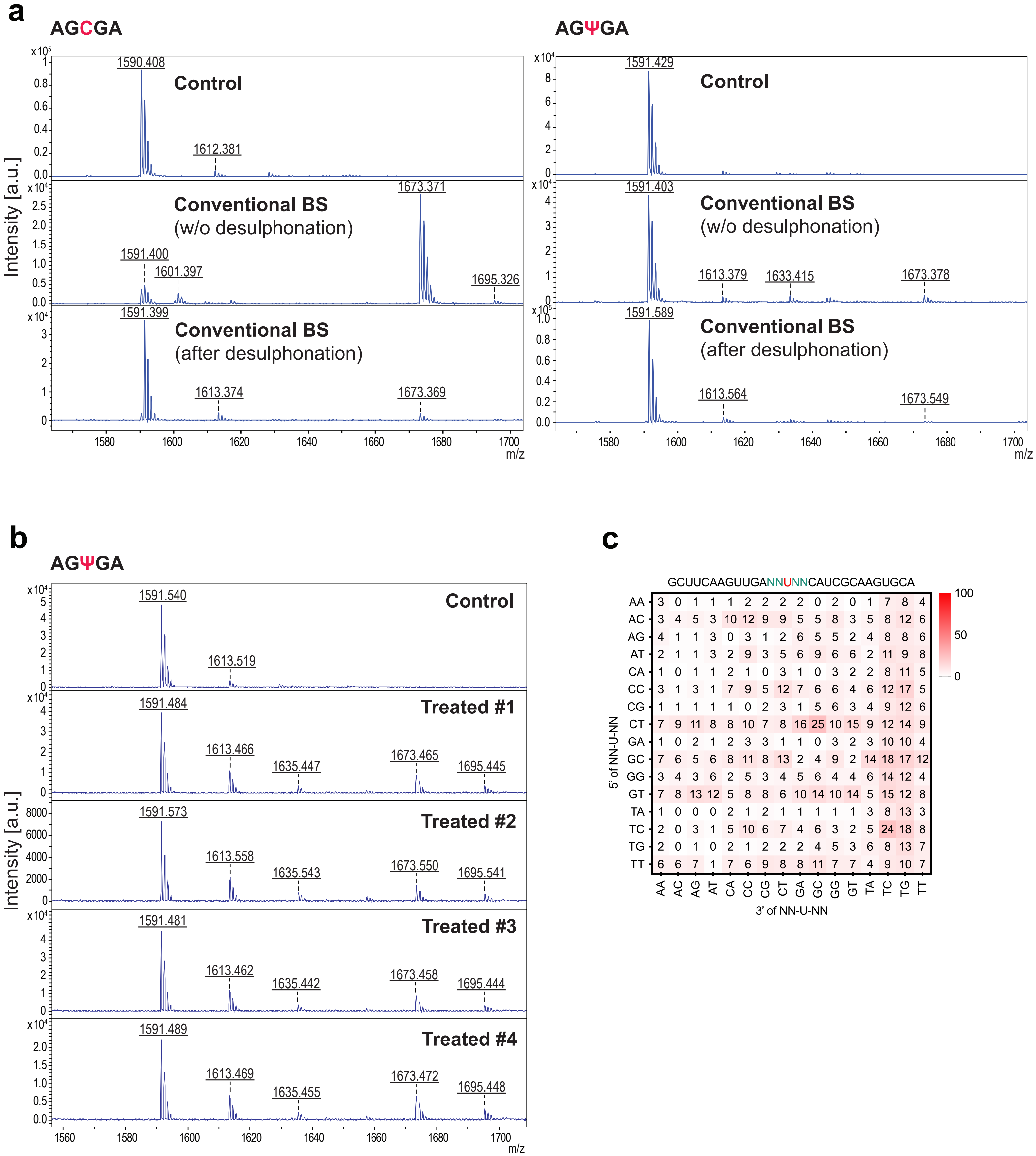

Supplementary Fig. 1 | BID-seq selectively converts Ψ into the Ψ-BS adduct without affecting C or U in RNA. **a**, Conventional bisulfite treatment converts cytidine (C) into uridine (U) but shows very low reactivity with pseudouridine (Ψ). **b**, Bisulfite treatment in RBS-seq converts a small portion of Ψ into the Ψ-BS adduct. **c**, Heatmap plot for deletion ratios on 256 motifs (NNUNN) after the optimized bisulfite treatment in BID-seq.

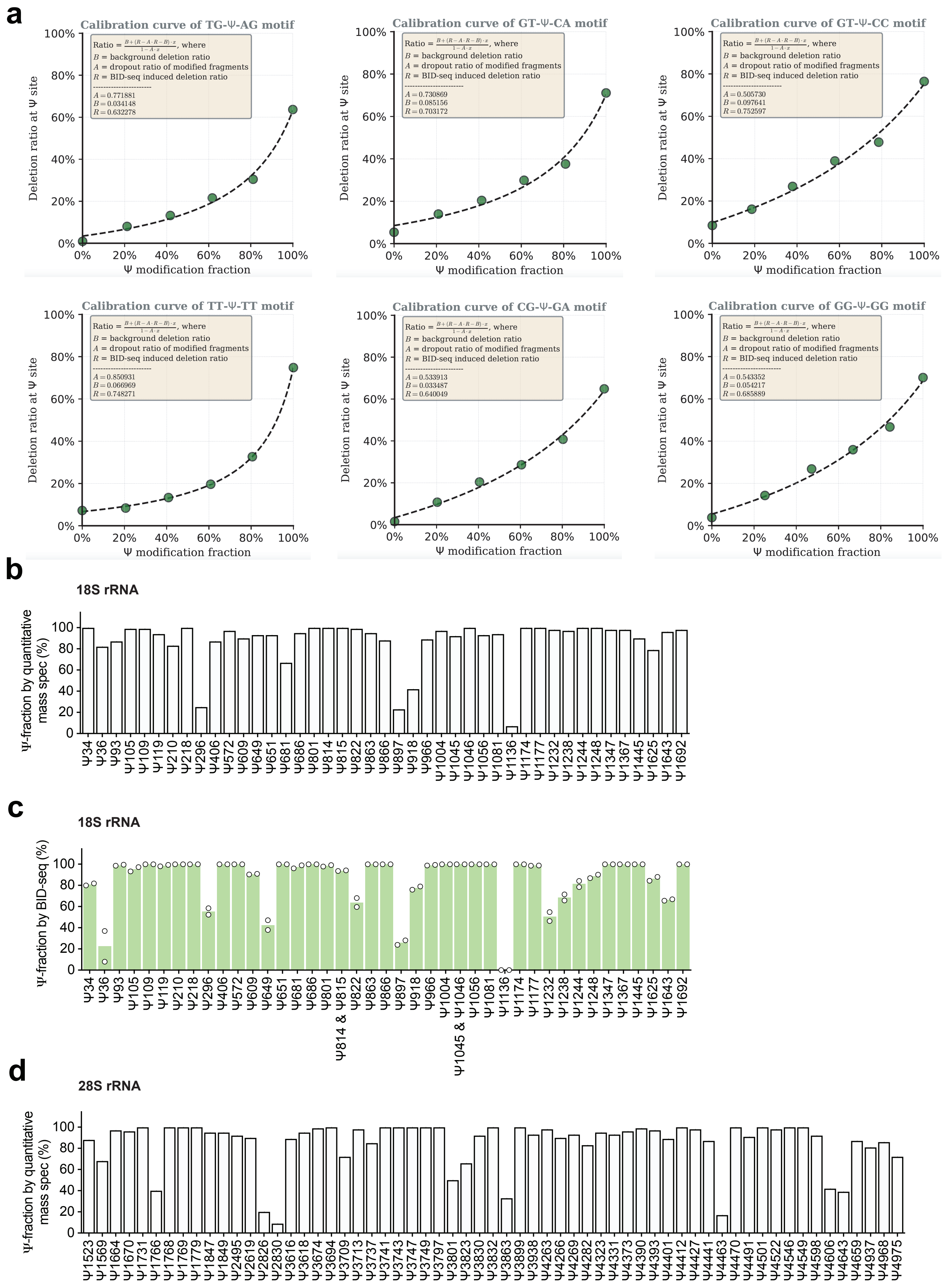

a
Calibration curve of TG-Ψ-AG motif
Ratio = (B+(R−A·R−B)·x)/(1−A·x), where
B = background deletion ratio
A = dropout ratio of modified fragments
R = BID-seq induced deletion ratio
A = 0.771881
B = 0.034148
R = 0.632278
Calibration curve of GT-Ψ-CA motif
A = 0.730869
B = 0.085156
R = 0.703172
Calibration curve of GT-Ψ-CC motif
A = 0.505730
B = 0.097641
R = 0.752597
Calibration curve of TT-Ψ-TT motif
A = 0.850931
B = 0.066969
R = 0.748271
Calibration curve of CG-Ψ-GA motif
A = 0.533913
B = 0.033487
R = 0.640049
Calibration curve of GG-Ψ-GG motif
A = 0.543352
B = 0.054217
R = 0.685889
Deletion ratio at Ψ site
Ψ modification fraction
b
18S rRNA
Ψ-fraction by quantitative mass spec (%)
c
18S rRNA
Ψ-fraction by BID-seq (%)
Ψ814 & Ψ815
Ψ1045 & Ψ1046
d
28S rRNA
Ψ-fraction by quantitative mass spec (%)

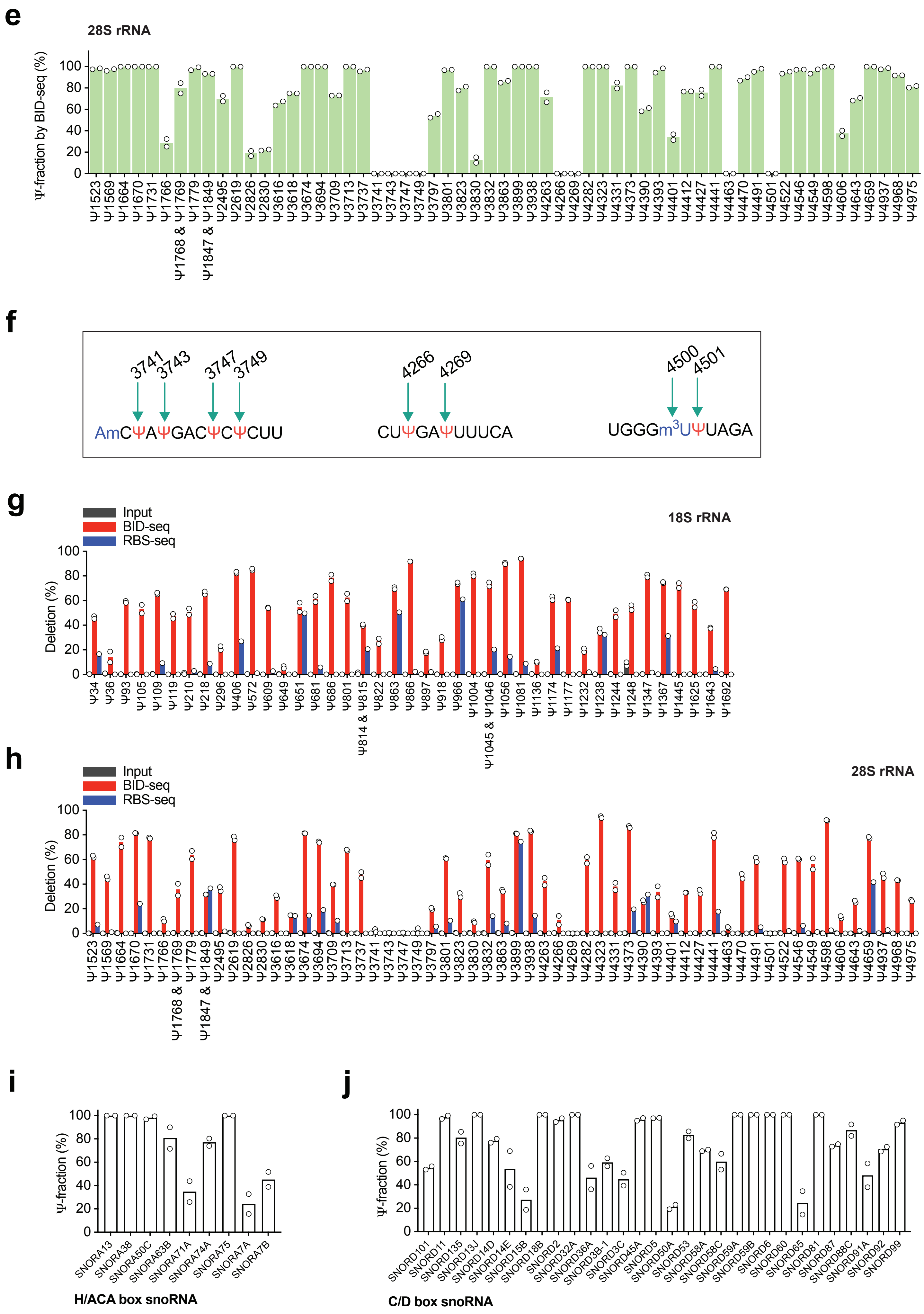

e
28S rRNA
Ψ-fraction by BID-seq (%)
0
20
40
60
80
100
Ψ1523 Ψ1569 Ψ1664 Ψ1670 Ψ1731 Ψ1766 Ψ1768 & Ψ1769 Ψ1779 Ψ1847 & Ψ1849 Ψ2495 Ψ2619 Ψ2826 Ψ2830 Ψ3616 Ψ3618 Ψ3674 Ψ3694 Ψ3709 Ψ3713 Ψ3737 Ψ3741 Ψ3743 Ψ3747 Ψ3749 Ψ3797 Ψ3801 Ψ3823 Ψ3830 Ψ3832 Ψ3863 Ψ3899 Ψ3938 Ψ4263 Ψ4266 Ψ4269 Ψ4282 Ψ4323 Ψ4331 Ψ4373 Ψ4390 Ψ4393 Ψ4401 Ψ4412 Ψ4427 Ψ4441 Ψ4463 Ψ4470 Ψ4491 Ψ4501 Ψ4522 Ψ4546 Ψ4549 Ψ4598 Ψ4606 Ψ4643 Ψ4659 Ψ4937 Ψ4968 Ψ4975
f
3741
3743
3747
3749
AmCΨAΨGACΨCΨCUU
4266
4269
CUΨGAΨUUUCA
4500
4501
UGGGm3UΨUAGA
g
Input
BID-seq
RBS-seq
18S rRNA
Deletion (%)
0
20
40
60
80
100
Ψ34 Ψ36 Ψ93 Ψ105 Ψ109 Ψ119 Ψ210 Ψ218 Ψ296 Ψ406 Ψ572 Ψ609 Ψ649 Ψ651 Ψ681 Ψ686 Ψ801 Ψ814 & Ψ815 Ψ822 Ψ863 Ψ866 Ψ897 Ψ918 Ψ966 Ψ1004 Ψ1045 & Ψ1046 Ψ1056 Ψ1081 Ψ1136 Ψ1174 Ψ1177 Ψ1232 Ψ1238 Ψ1244 Ψ1248 Ψ1347 Ψ1367 Ψ1445 Ψ1625 Ψ1643 Ψ1692
h
Input
BID-seq
RBS-seq
28S rRNA
Deletion (%)
0
20
40
60
80
100
Ψ1523 Ψ1569 Ψ1664 Ψ1670 Ψ1731 Ψ1766 Ψ1768 & Ψ1769 Ψ1779 Ψ1847 & Ψ1849 Ψ2495 Ψ2619 Ψ2826 Ψ2830 Ψ3616 Ψ3618 Ψ3674 Ψ3694 Ψ3709 Ψ3713 Ψ3737 Ψ3741 Ψ3743 Ψ3747 Ψ3749 Ψ3797 Ψ3801 Ψ3823 Ψ3830 Ψ3832 Ψ3863 Ψ3899 Ψ3938 Ψ4263 Ψ4266 Ψ4269 Ψ4282 Ψ4323 Ψ4331 Ψ4373 Ψ4390 Ψ4393 Ψ4401 Ψ4412 Ψ4427 Ψ4441 Ψ4463 Ψ4470 Ψ4491 Ψ4501 Ψ4522 Ψ4546 Ψ4549 Ψ4598 Ψ4606 Ψ4643 Ψ4659 Ψ4937 Ψ4968 Ψ4975
i
Ψ-fraction (%)
0
20
40
60
80
100
SNORA13 SNORA38 SNORA50C SNORA63B SNORA71A SNORA74A SNORA75 SNORA7A SNORA7B
H/ACA box snoRNA
j
Ψ-fraction (%)
0
20
40
60
80
100
SNORD101 SNORD11 SNORD135 SNORD13J SNORD14D SNORD14E SNORD15B SNORD18B SNORD2 SNORD32A SNORD36A SNORD3B-1 SNORD3C SNORD45A SNORD5 SNORD50A SNORD53 SNORD58A SNORD58C SNORD59A SNORD59B SNORD6 SNORD60 SNORD65 SNORD81 SNORD87 SNORD88C SNORD91A SNORD92 SNORD99
C/D box snoRNA

Supplementary Fig. 2 | BID-seq validated known Ψ sites in human rRNA. **a**, Representative sequence-context-dependent calibration curves for Ψ fraction quantification. **b**, The Ψ fraction of known Ψ sites in HeLa 18S rRNA by mass spectrometry. **c**, The Ψ fraction of known Ψ sites in HeLa 18S rRNA by BID-seq. n = 2, biologically independent samples. **d**, The Ψ fraction of known Ψ sites in HeLa 28S rRNA by mass spectrometry. **e**, The Ψ fraction of known Ψ sites in HeLa 28S rRNA by BID-seq. n = 2, biologically independent samples. **f**, The surrounding rRNA sequences for 28S rRNA Ψ3741/Ψ3743/Ψ3747/Ψ3749, 28S rRNA Ψ4266/Ψ4269, and 28S rRNA Ψ4501. **g**, The deletion ratios of all known Ψ sites in HeLa 18S rRNA, comparing BID-seq treatment with RBS-seq treatment and the input. **h**, The deletion ratios of all known Ψ sites in HeLa 28S rRNA, comparing BID-seq treatment with RBS-seq treatment and the input. **i**, Ψ modification fraction of Ψ sites detected in H/ACA box snoRNA when applying BID-seq to HeLa small RNAs (< 200 nt). **j**, Ψ modification fraction of Ψ sites detected in C/D box snoRNA when applying BID-seq to HeLa small RNAs (< 200 nt). For **g**-**j**, n = 2, biologically independent samples.

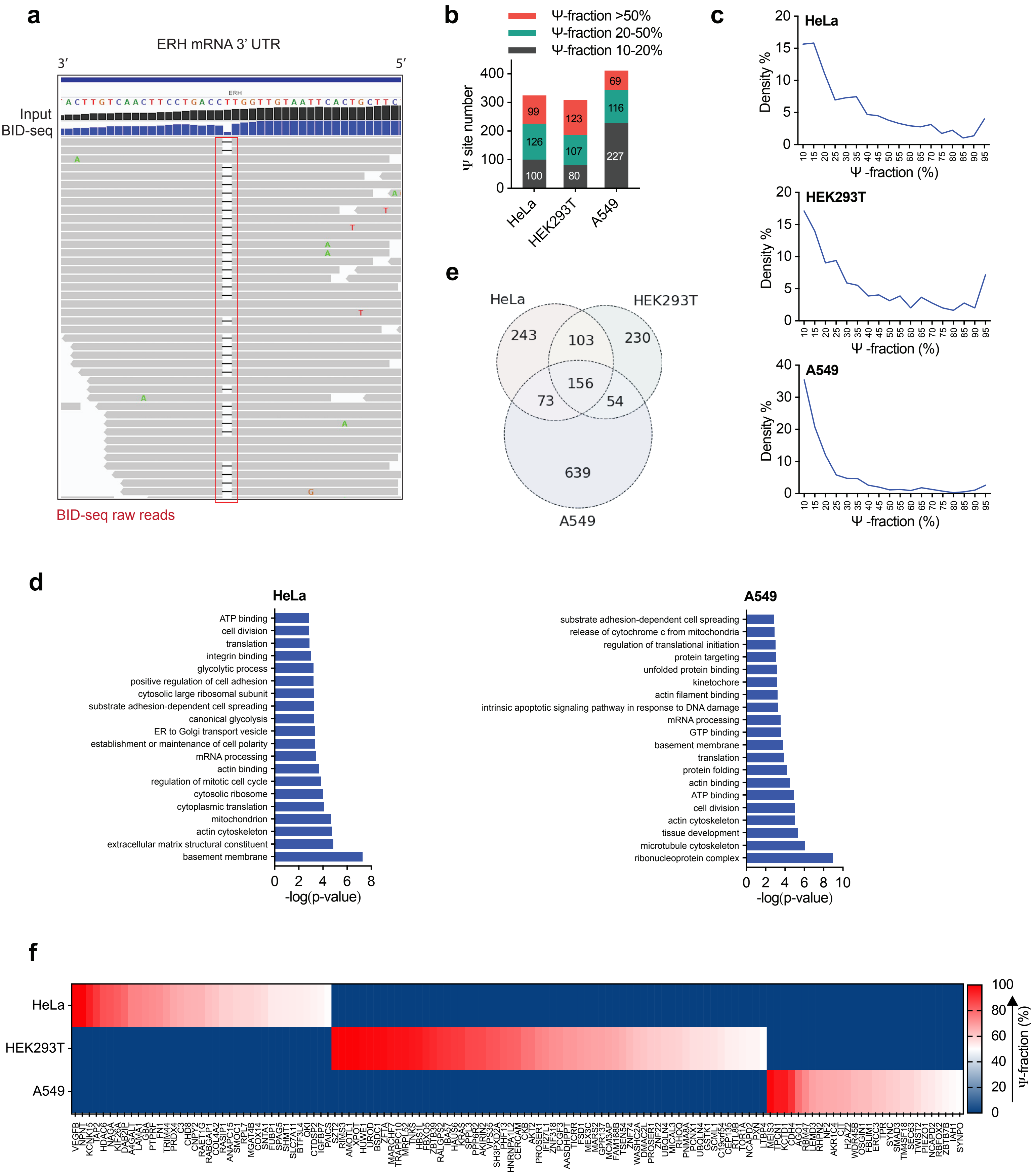
a
ERH mRNA 3′ UTR
3′
5′
Input
BID-seq
BID-seq raw reads
b
Ψ-fraction >50%
Ψ-fraction 20-50%
Ψ-fraction 10-20%
Ψ site number
HeLa
HEK293T
A549
c
HeLa
HEK293T
A549
Density %
Ψ -fraction (%)
e
HeLa
HEK293T
A549
243
103
230
156
73
54
639
d
HeLa
A549
-log(p-value)
ATP binding
cell division
translation
integrin binding
glycolytic process
positive regulation of cell adhesion
cytosolic large ribosomal subunit
substrate adhesion-dependent cell spreading
canonical glycolysis
ER to Golgi transport vesicle
establishment or maintenance of cell polarity
mRNA processing
actin binding
regulation of mitotic cell cycle
cytosolic ribosome
cytoplasmic translation
mitochondrion
actin cytoskeleton
extracellular matrix structural constituent
basement membrane
substrate adhesion-dependent cell spreading
release of cytochrome c from mitochondria
regulation of translational initiation
protein targeting
unfolded protein binding
kinetochore
actin filament binding
intrinsic apoptotic signaling pathway in response to DNA damage
mRNA processing
GTP binding
basement membrane
translation
protein folding
actin binding
ATP binding
cell division
actin cytoskeleton
tissue development
microtubule cytoskeleton
ribonucleoprotein complex
f
HeLa
HEK293T
A549
Ψ-fraction (%)

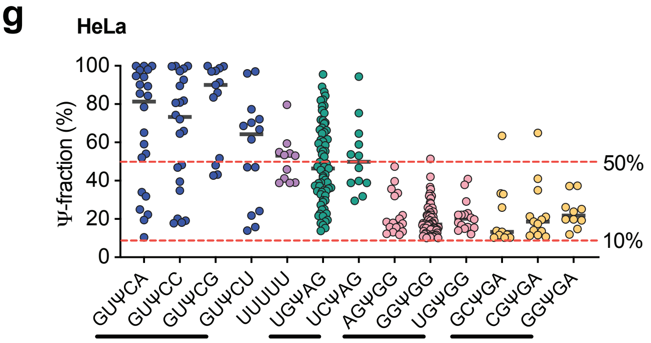

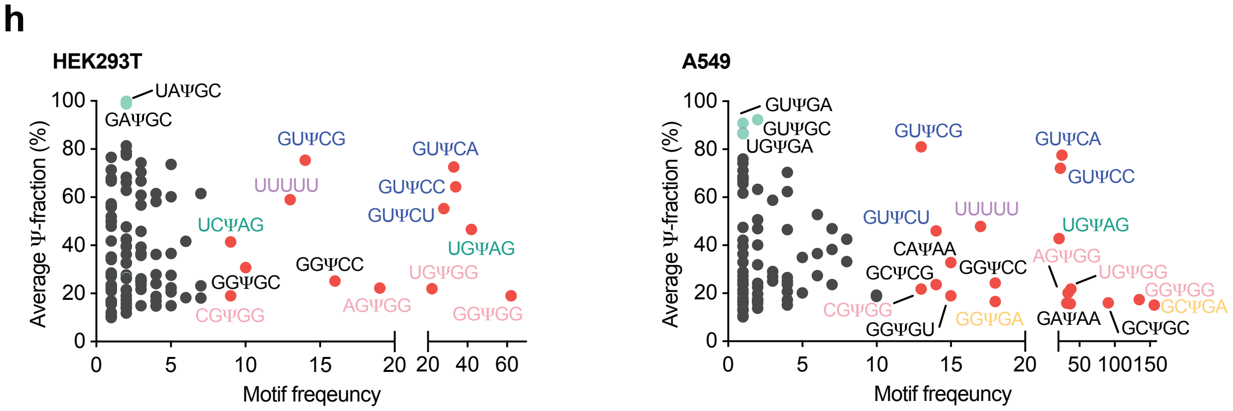

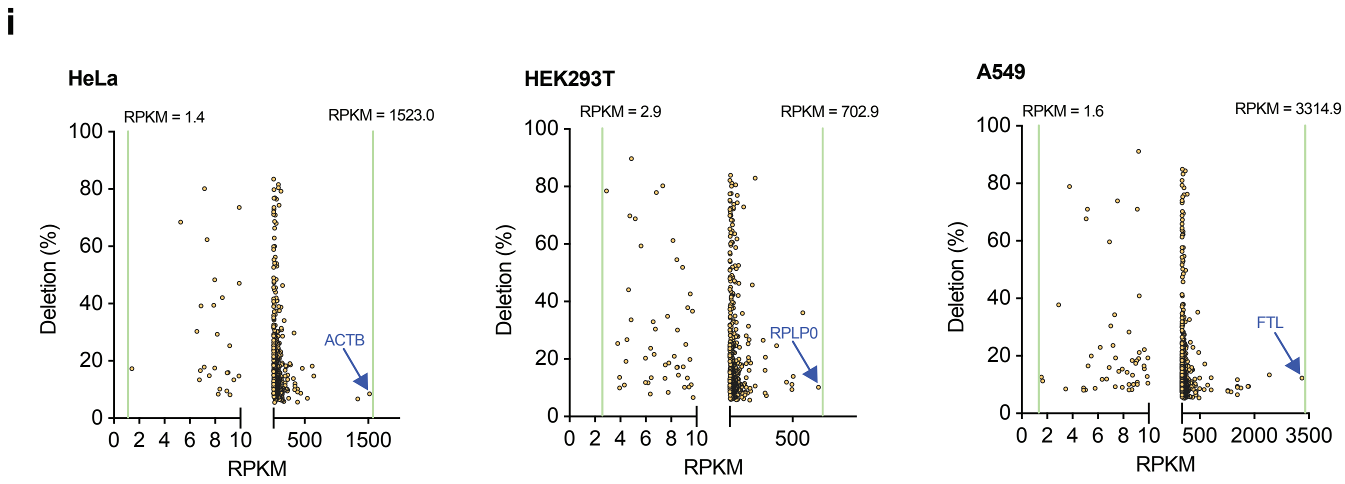

Supplementary Fig. 3 | BID-seq reveals Ψ sites in mRNAs of HeLa, HEK293T and A549 cells. **a**, An example IGV plot to show raw reads coverage at the highly modified Ψ site within 3' UTR of HeLa ERH mRNA, where all deletion signatures occur at internal positions within the reads. **b**, BID-seq reveals 325, 310, and 412 Ψ sites (modification fraction above 10%, deletion count above 10) in HeLa, HEK293T and A549 cells, respectively. **c**, The mRNA Ψ site number distribution normalized to Ψ-fraction bins, in three human cell lines. **d**, Top 20 enriched GO clusters from Ψ-modified genes carrying mRNA Ψ, in HeLa and A549 cells, respectively. One-sided Fisher's Exact test. Adjusted p-values using the linear step-up method. **e**, The pie chart to show overlapped Ψ sites above 10% Ψ-fraction in three cell lines. **f**, The heatmap plot of 127 cell-line-specific highly modified Ψ sites (above 50% Ψ-fraction) in the corresponding gene name versus each cell line. **g**, The modification level distribution of several most frequent Ψ motif contexts on HeLa mRNA. n = 22, 22, 13, 14, 11, 69, 12, 17, 61, 16, 11, 13 and 10 independent Ψ sites within GUΨCA, GUΨCC, GUΨCG, GUΨCU, UUUUU, UGΨAG, UCΨAG, AGΨGG, GGΨGG, UGΨGG, GCΨGA, CGΨGA and GGΨGA motifs, respectively. **h**, Distribution of motifs for 543 and 922 Ψ sites in HEK293T and A549 mRNA, respectively, with "X axis" as the motif frequency and "Y axis" showing the average Ψ modification fraction of each motif. **i**, The correlation of deletion ratio at each Ψ site versus RPKM value of the corresponding mRNA, in HeLa, HEK293T and A549 cells, respectively.

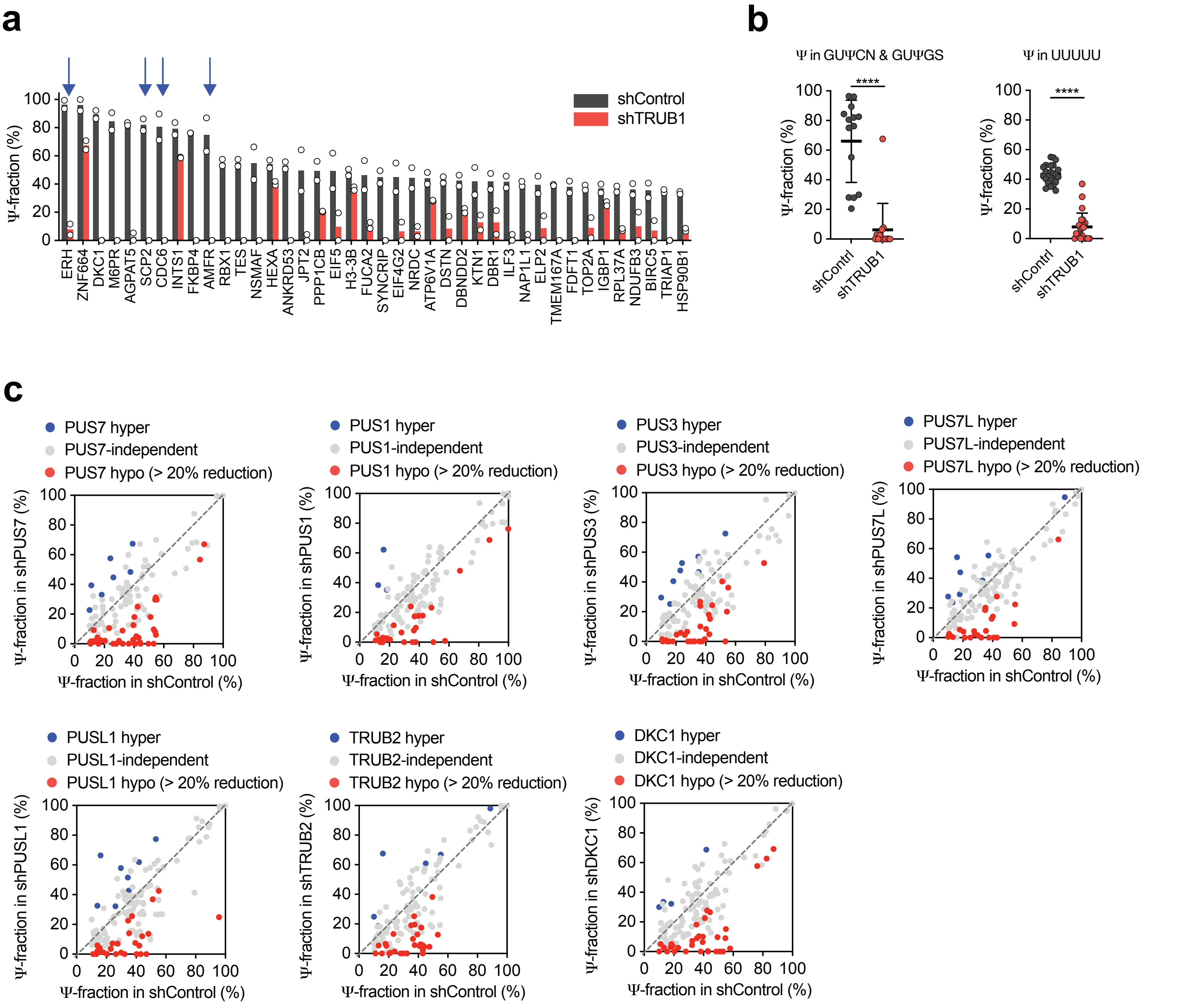

Supplementary Fig. 4 | BID-seq assigns 'writer' proteins for mRNA Ψ sites. a, The modification fraction of top 40 TRUB1-installed Ψ sites in HeLa mRNA, in shTRUB1 versus shControl. ERH, SCP2, AMFR and CDC6 are marked by blue arrows. n = 2, biologically independent samples. **b**, TRUB1-insatlled Ψ sites mostly appear within the GUΨCN, GUΨGS (S = C or G) and poly-U (UUUUU) motifs, in HeLa mRNA. Left: n = 14 independent Ψ sites within GUΨCN and GUΨGS motifs (N = A or C or G or U; S = G or C); ****P< 0.0001; paired, two-tailed t-test. Right: n = 24 independent Ψ sites within UUUUU motif; ****P< 0.0001; paired, two-tailed t-test. Data are presented as mean values ± s.d. **c**, Among 133 Ψ sites (above 10% Ψ-fraction) in shControl HeLa mRNA, scatter plot of BID-seq data showing the reduced Ψ-fraction at 40, 28, 30, 24, 28, 28, and 33 Ψ sites in PUS1-, PUS7-, PUS3-, PUS7L-, PUSL1-, TRUB2-, and DKC1-depleted cells, respectively.

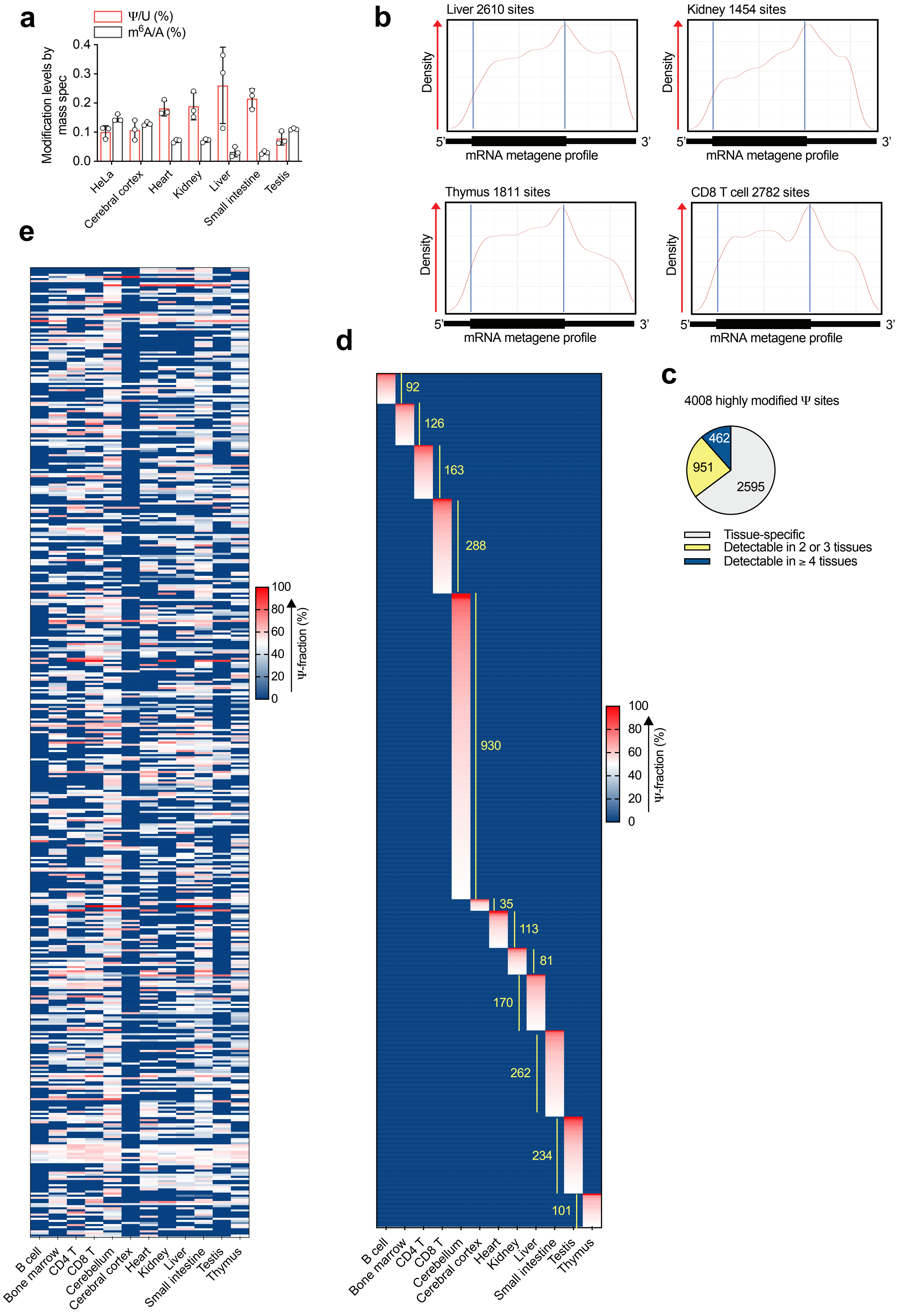
a
Ψ/U (%)
m6A/A (%)
Modification levels by mass spec
0.4
0.3
0.2
0.1
0.0
HeLa
Cerebral cortex
Heart
Kidney
Liver
Small intestine
Testis
b
Liver 2610 sites
Kidney 1454 sites
Thymus 1811 sites
CD8 T cell 2782 sites
Density
5'
3'
mRNA metagene profile
c
4008 highly modified Ψ sites
462
951
2595
Tissue-specific
Detectable in 2 or 3 tissues
Detectable in ≥ 4 tissues
d
92
126
163
288
930
35
113
81
170
262
234
101
100
80
60
40
20
0
Ψ-fraction (%)
B cell
Bone marrow
CD4 T
CD8 T
Cerebellum
Cerebral cortex
Heart
Kidney
Liver
Small intestine
Testis
Thymus
e
100
80
60
40
20
0
Ψ-fraction (%)
B cell
Bone marrow
CD4 T
CD8 T
Cerebellum
Cerebral cortex
Heart
Kidney
Liver
Small intestine
Testis
Thymus

Supplementary Fig. 5 | BID-seq detects hundreds to thousands of highly modified Ψ sites in mRNA from multiple mouse tissues. **a**, The modification level (by LC-MS/MS) of Ψ/U and m6A/A in mRNA isolated from mouse cerebral cortex, heart, kidney, liver, small intestine and testis, respectively, compared with that in HeLa cells. n = 3, biologically independent samples; data are presented as mean values ± s.d. **b**, The metagene plot of 2,610, 1,454, 1,811 and 2,782 Ψ sites (modification fraction above 10%) in mRNA from mouse liver, kidney, thymus and CD8 T cell, respectively. **c**, Pie chart showing the site number distribution for 4,008 highly modified Ψ sites (above 50% Ψ fraction) from mouse tissue mRNA, as tissue-specific highly modified Ψ, highly modified Ψ in at least 1 tissue & detectable (above 10% Ψ fraction) in 2 or 3 tissues, and highly modified Ψ in at least 1 tissue & detectable (above 10% Ψ fraction) in above 4 tissues. **d**, The heatmap plot of Ψ-fraction for 2,595 tissue-specific highly modified Ψ sites, in a matrix of the corresponding gene name versus each tissue type. **e**, The heatmap plot of Ψ-fraction for 462 highly modified Ψ sites showing above 50% Ψ-fraction in at least 1 tissue and detectable (above 10% Ψ fraction) in above 4 tissues, in a matrix of the corresponding gene name versus each tissue type.

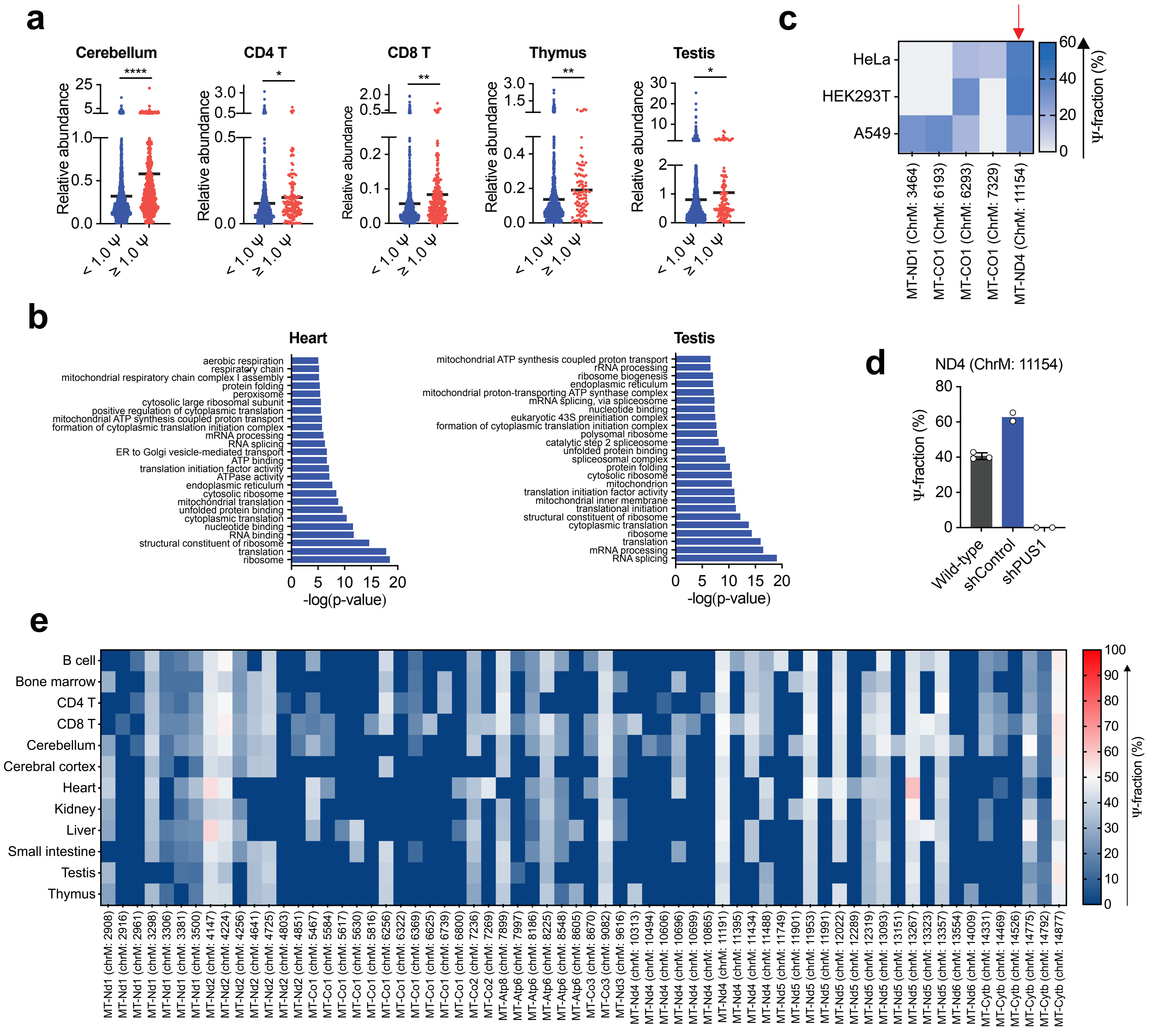

Supplementary Fig. 6 | mRNA Ψ affects mRNA abundance in multiple mouse tissues. **a**, The normalized mRNA abundance of Ψ moderate-strength genes (< 1.0) vs. Ψ high-strength genes (above 1.0) in mouse cerebellum, CD4 T cells, CD8 T cells, thymus and testis. The Ψ-strength of each gene was defined as the sum of Ψ fraction at all the Ψ sites within one gene. n = 1,997 Ψ-moderate-strength genes and n = 746 Ψ-high-strength genes, in cerebellum; n = 843 Ψ-moderate-strength genes and n = 149 Ψ-high-strength, in CD4 T cells; n = 1,022 Ψ-moderate-strength genes and n = 273 Ψ-high-strength genes, in CD8 T cells; n = 1,018 Ψ-moderate-strength genes and n = 98 Ψ-high-strength genes, in thymus; n = 1,356 Ψ-moderate-strength genes and n = 124 Ψ-high-strength genes, in testis. *P* = <0.0001, 0.0279, 0.0012, 0.0026, 0.0313, respectively, in five mouse tissues; unpaired, two-tailed t-test; **P* < 0.05; ***P* < 0.01; ****P* < 0.001; and *****P* < 0.0001. **b**, Top 25 enriched GO clusters from non-tissue-specific Ψ-modified genes, in mouse heart and testis, respectively. One-sided Fisher's Exact test. Adjusted p-values using the linear step-up method. **c**, The heatmap plot of Ψ fraction for Ψ sites within mitochondria-encoded mRNA in 3 human cell lines, in a matrix of Ψ site location versus each cell line. **d**, The Ψ modification fraction of mt-ND4 mRNA Ψ site in wild-type HeLa cells, shControl HeLa cells and PUS1-depleted HeLa cells, respectively. For wild-type HeLa cells, n = 3, biologically independent samples; data are presented as mean values ± s.d. For shControl and PUS1-depleted HeLa cells, n = 2, biologically independent samples. **e**, The heatmap plot of Ψ-fraction for 66 Ψ sites within mitochondrial mRNA in mouse tissues, in a matrix of Ψ site location versus tissue type.

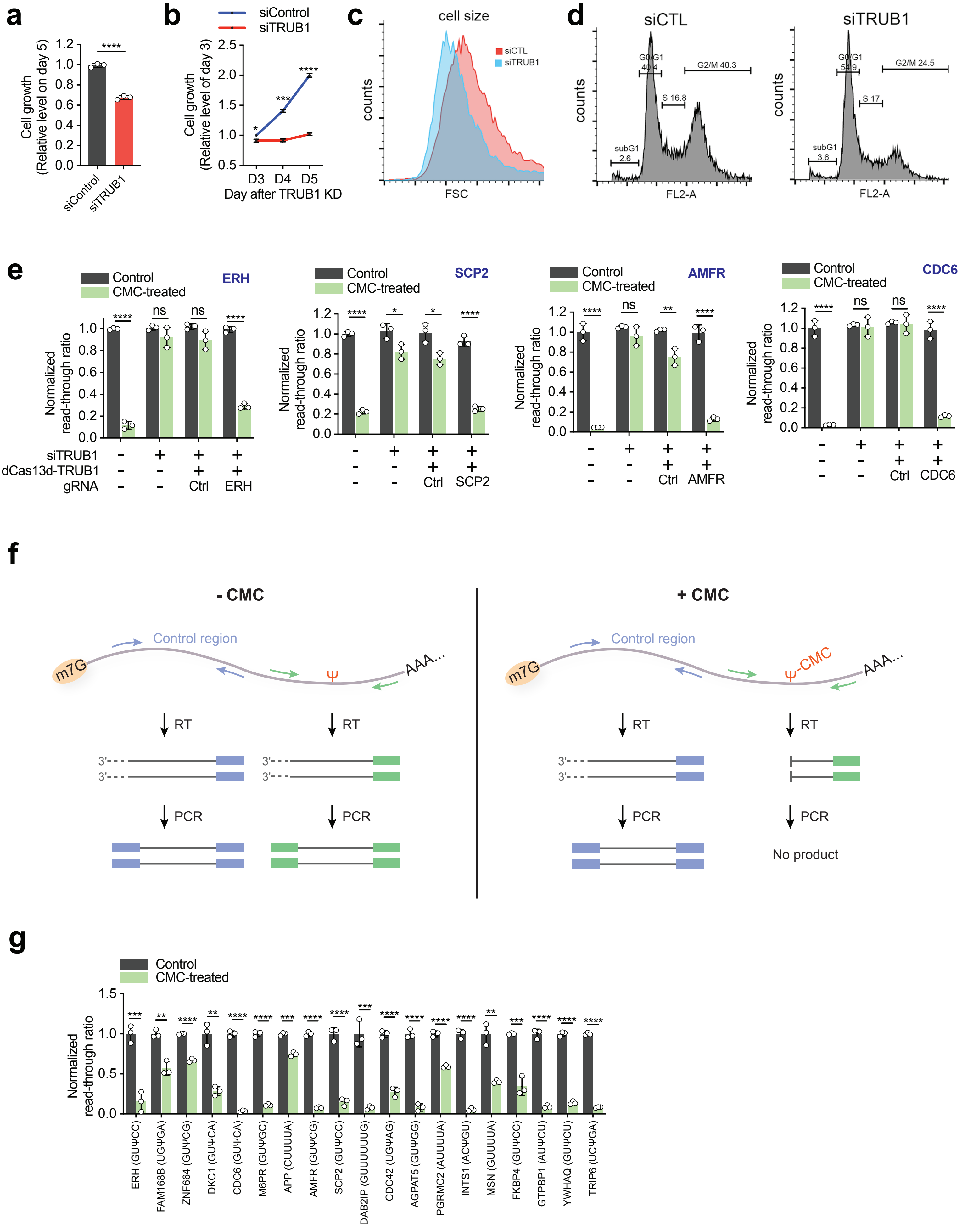
a
Cell growth (Relative level on day 5)
siControl
siTRUB1
b
siControl
siTRUB1
Cell growth (Relative level of day 3)
D3 D4 D5
Day after TRUB1 KD
c
cell size
siCTL
siTRUB1
counts
FSC
d
siCTL
siTRUB1
counts
FL2-A
e
Control
CMC-treated
ERH
SCP2
AMFR
CDC6
Normalized read-through ratio
siTRUB1
dCas13d-TRUB1
gRNA
Ctrl
f
- CMC
+ CMC
Control region
m7G
AAA...
Ψ
Ψ-CMC
RT
PCR
No product
g
Control
CMC-treated
Normalized read-through ratio
ERH (GUΨCC)
FAM168B (UGΨGA)
ZNF664 (GUΨCG)
DKC1 (GUΨCA)
CDC6 (GUΨCA)
M6PR (GUΨGC)
APP (CUUUUA)
AMFR (GUΨCG)
SCP2 (GUΨCC)
DAB2IP (GUUUUUUG)
CDC42 (UGΨAG)
AGPAT5 (GUΨGG)
PGRMC2 (AUUUUA)
INTS1 (ACΨGU)
MSN (GUUUUA)
FKBP4 (GUΨCC)
GTPBP1 (AUΨCU)
YWHAQ (GUΨCU)
TRIP6 (UCΨGA)

h

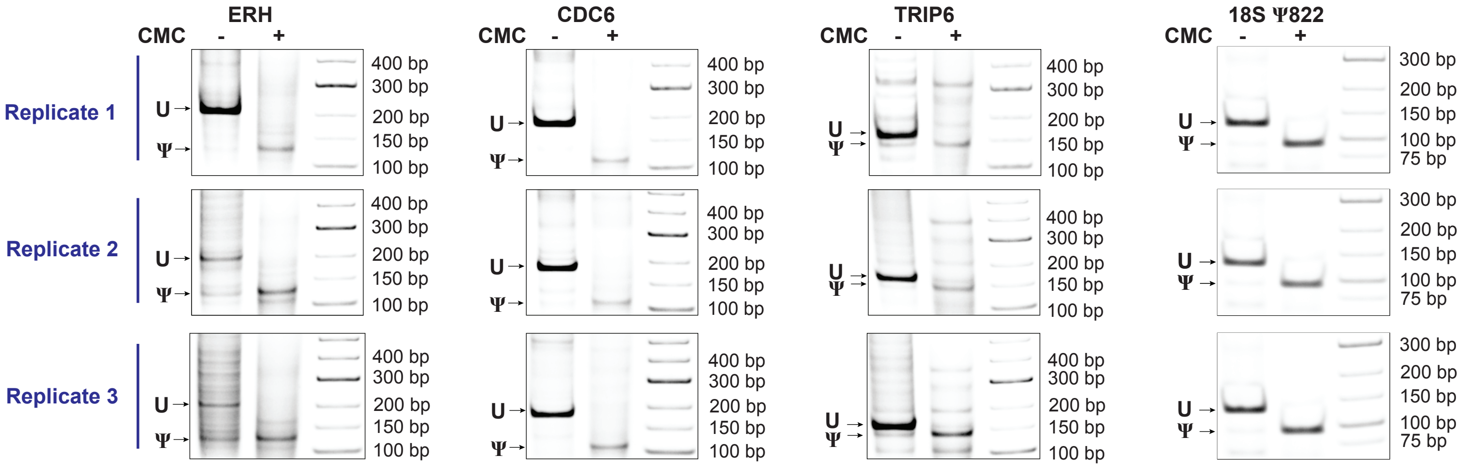

i

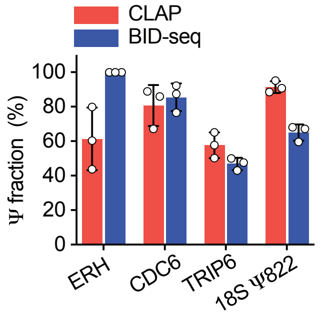

j

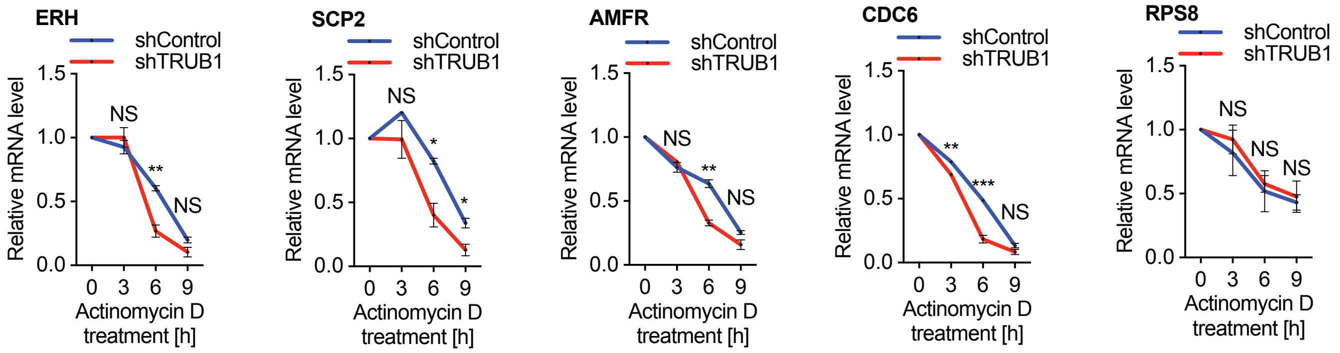

Supplementary Fig. 7 | TRUB1 depletion impacts cell growth and affects mRNA stability. a, TRUB1 depletion inhibit cell growth. Growth of HeLa cells was measured by SRB assay on day 5 after transfection with corresponding siRNA. ****P < 0.0001; unpaired, two-tailed t-test. **b**, TRUB1 depletion inhibits cell growth. Cell growth of HeLa cells transfected with siRNA was measured by SRB assay from Day 3 to Day 5. P = 0.0368, 0.0001, <0.0001, respectively, for the three time points; unpaired, two-tailed t-test. **c**, TRUB1 depletion decreases cell size, compared with siControl. **d**, TRUB1 depletion leads to G1 phase arrest, after 72-hour siRNA knockdown. **e**, Validation of mRNA Ψ site by CMC-assisted RT-qPCR assays, in siTRUB1 versus siControl. Then the restoration of Ψ was performed by dCas13d-TRUB1 delivery. The normalized read-through ratios were measured in untreated and CMC-treated samples by designed RT-qPCR primers. For ERH, P = <0.0001, 0.1748, 0.0663, <0.0001, respectively; For SCP2, P = <0.0001, 0.0264, 0.0148, <0.0001, respectively; For AMFR, P = <0.0001, 0.1776, 0.0054, <0.0001, respectively; For CDC6, P = <0.0001, 0.7442, 0.8054, <0.0001, respectively; unpaired, two-tailed t-test. **f**, A flowchart of CMC-based RT-qPCR assay to quantify read-through ratios (the CMC-modified Ψ blocks RT and the following qPCR amplification). **g**, CMC-based RT-qPCR assay verified an array of Ψ sites within different sequence motifs. For the 19 Ψ sites, P = 0.0009, 0.0017, <0.0001, 0.0011, <0.0001, <0.0001, 0.0002, <0.0001, <0.0001, 0.0006, <0.0001, <0.0001, <0.0001, <0.0001, 0.0014, 0.0006, <0.0001, <0.0001, <0.0001, respectively; unpaired, two-tailed t-test. **h**, Ψ site validation through "CMC-RT and Ligation Assisted PCR analysis of Ψ modification" (CLAP). CLAP assay identified four Ψ sites in ERH, CDC6, TRIP6, and 18S rRNA Ψ822, respectively, as bands of expected sizes on gel images. **i**, The estimated Ψ stoichiometry for four Ψ sites in ERH, CDC6, TRIP6, and 18S rRNA Ψ822, respectively, by CLAP assay versus BID-seq. **j**, RT-qPCR assays validating the decreased mRNA half-life when silencing TRUB1 in HeLa cells, for TRUB1-targets carrying a highly modified Ψ site in mRNA of ERH, SCP2, AMFR, and CDC6, compared with RPS8 as the non-target (without detectable Ψ in its mRNA). For ERH, P = 0.4689, 0.0032, 0.0941, respectively; For SCP2, P = 0.2284, 0.0117, 0.0233, respectively; For AMFR, P = 0.3334, 0.0012, 0.0825, respectively; For CDC6, P = 0.0049, 0.0006, 0.1418, respectively; For RPS8, P = 0.6453, 0.7616, 0.7601, respectively; unpaired, two-tailed t-test. For **a**, **b**, **e**, **g**, **i**, and **j**, n = 3, biologically independent samples; data are presented as mean values ± s.d.; NS, $P \geq 0.05$; *P < 0.05; **P < 0.01; ***P < 0.001; and ****P < 0.0001.

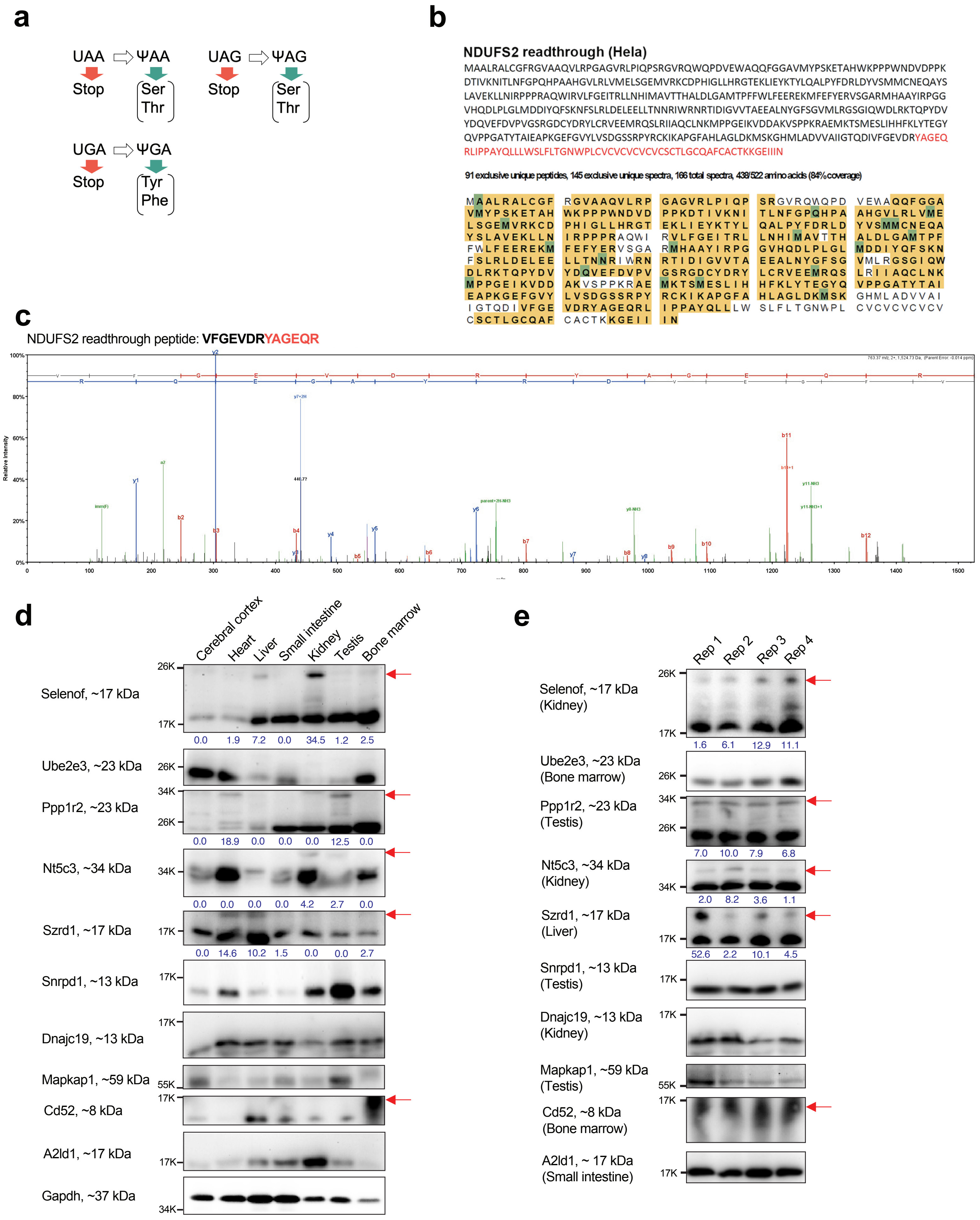

Supplementary Fig. 8 | Ψ promotes the stop codon read-through in HeLa cells and mouse tissues. **a**, The presence of Ψ within stop codons can convert stop codons into sense codons and promote read-through. **b**, Mass spectrometry captured the read-through peptide fragment across the NDUFS2 stop codon in HeLa cells. **c**, NDUFS2 read-through peptide (red part in the upper panel) was detected by mass spectrometry. **d**, The stop codon read-through for 10 selected proteins, by immunoblotting (IB) to test them in seven different mouse tissues. **e**, The band shifts in four different mice repeats. Some individual variations were observed for *Selenof, Ppp1r2, Nt5c3*, and *Szrd1*. For **d**-**e**, the percentage numbers of read-through ratio are shown; the readthrough protein bands are labeled by red arrows.
