# Supplementary material for: Quantitative sequencing using BID-seq uncovers abundant pseudouridines in mammalian mRNA at base resolution
Source: Nat Biotechnol. 2022 Oct 27;41(3):344–54. doi: 10.1038/s41587-022-01505-w (PMC10017504; doi:10.1038/s41587-022-01505-w)
Supplement: Supplementary file 2 — Reporting Summary [file 41587_2022_1505_MOESM2_ESM.pdf]

nature research

Corresponding author(s): Chuan He, Qing Dai, Li-Sheng Zhang

Last updated by author(s): Sep 6, 2022

Reporting Summary

Nature Research wishes to improve the reproducibility of the work that we publish. This form provides structure for consistency and transparency in reporting. For further information on Nature Research policies, see our Editorial Policies and the Editorial Policy Checklist.

Statistics

For all statistical analyses, confirm that the following items are present in the figure legend, table legend, main text, or Methods section.

n/a	Confirmed	
☐	☒	The exact sample size (*n*) for each experimental group/condition, given as a discrete number and unit of measurement
☐	☒	A statement on whether measurements were taken from distinct samples or whether the same sample was measured repeatedly
☐	☒	The statistical test(s) used AND whether they are one- or two-sided<br>*Only common tests should be described solely by name; describe more complex techniques in the Methods section.*
☒	☐	A description of all covariates tested
☒	☐	A description of any assumptions or corrections, such as tests of normality and adjustment for multiple comparisons
☐	☒	A full description of the statistical parameters including central tendency (e.g. means) or other basic estimates (e.g. regression coefficient) AND variation (e.g. standard deviation) or associated estimates of uncertainty (e.g. confidence intervals)
☐	☒	For null hypothesis testing, the test statistic (e.g. *F*, *t*, *r*) with confidence intervals, effect sizes, degrees of freedom and *P* value noted<br>*Give P values as exact values whenever suitable.*
☒	☐	For Bayesian analysis, information on the choice of priors and Markov chain Monte Carlo settings
☒	☐	For hierarchical and complex designs, identification of the appropriate level for tests and full reporting of outcomes
☐	☒	Estimates of effect sizes (e.g. Cohen's *d*, Pearson's *r*), indicating how they were calculated

Our web collection on statistics for biologists contains articles on many of the points above.

Software and code

Policy information about availability of computer code

Data collection	All libraries were sequenced on Illumina NovaSeq 6000 with single-end 100 bp read length.
Data analysis	Tophat2 (version 2.1.1), Bowtie2 (version 2.4.0), bam-readcount (version 0.8.0), cutadapt (version 1.15), BBMap (version 38.73), samtools (version 1.9), Cufflinks (version 2.2.1), and IGV software (version 2.8.0) were used to analyze the RNA-seq data. Prism (version 9.2.0) and ImageJ (version 1.53a) were used for data plot.

For manuscripts utilizing custom algorithms or software that are central to the research but not yet described in published literature, software must be made available to editors and reviewers. We strongly encourage code deposition in a community repository (e.g. GitHub). See the Nature Research guidelines for submitting code & software for further information.

Data

Policy information about availability of data

All manuscripts must include a data availability statement. This statement should provide the following information, where applicable:

- Accession codes, unique identifiers, or web links for publicly available datasets
- A list of figures that have associated raw data
- A description of any restrictions on data availability

The BID-seq data generated by this study have been deposited in NCBI Gene Expression Omnbus (GEO) under the accession number GSE179798.

Field-specific reporting

Please select the one below that is the best fit for your research. If you are not sure, read the appropriate sections before making your selection.

☒ Life sciences ☐ Behavioural & social sciences ☐ Ecological, evolutionary & environmental sciences

For a reference copy of the document with all sections, see nature.com/documents/nr-reporting-summary-flat.pdf

Life sciences study design

All studies must disclose on these points even when the disclosure is negative.

Sample size	No statistical methods were used to predetermine sample size. For sequencing data, we collected data from two or three biological replicates. Sample size were determined based on our prior experience on similar experiments (Nat. Biotechnol. 40(8), 1210-1219 (2022); Nat. Methods 16(12), 1281-1288 (2019)).
Data exclusions	No data were excluded from analysis.
Replication	Two or three biologically independent replicates were performed independently. All attempts were successful.
Randomization	Samples in this study were not randomized. We did not set up the control for covariates in the animal experiments because all groups were age and sex matched.
Blinding	Blinding was not used for this study because cell culture, sample preparation, reagents, experimental settings were kept consistent for each experiment. The key experiments in this study were conducted by several lab members independently and gave the similar results.

Reporting for specific materials, systems and methods

We require information from authors about some types of materials, experimental systems and methods used in many studies. Here, indicate whether each material, system or method listed is relevant to your study. If you are not sure if a list item applies to your research, read the appropriate section before selecting a response.

Materials & experimental systems

n/a	Involved in the study	
☐	☒	Antibodies
☐	☒	Eukaryotic cell lines
☒	☐	Palaeontology and archaeology
☐	☒	Animals and other organisms
☒	☐	Human research participants
☒	☐	Clinical data
☒	☐	Dual use research of concern

Methods

n/a	Involved in the study	
☒	☐	ChIP-seq
☒	☐	Flow cytometry
☒	☐	MRI-based neuroimaging

Antibodies

Antibodies used	rabbit monoclonal anti-NDUFS2, clone EPR16266 (abcam, ab192022, 1:1000),<br>mouse monoclonal anti-GAPDH, clone 0411 (Santa Cruz, sc-47724, 1:1000),<br>rabbit polyclonal anti-SELENOF (My BioSource, MBS3208942, 1:500),<br>mouse monoclonal anti-UBE2E3, clone OTI7E8 (Novus Biologicals, NBP2-03819, 1:500),<br>rabbit polyclonal anti-PPP1R2 (ThermoFisher Scientific, PA5-115787, 1:500),<br>rabbit polyclonal anti-NT5C3 (Proteintech, 11393-1-AP, 1:500),<br>rabbit polyclonal anti-SZRD1 (ThermoFisher Scientific, A304-742A, 1:1000),<br>rabbit polyclonal anti-SNRPD1 (Novus Biologicals, NBP2-36427, 1:500),<br>rabbit polyclonal anti-DNAJC19 (ThermoFisher Scientific, PA5-98770, 1:1000),<br>rabbit polyclonal anti-MAPKAP1 (Proteintech, 15463-1-AP, 1:500),<br>rabbit monoclonal anti-CD52, clone EPR3153(2) (abcam, ab125071, 1:1000),<br>rabbit polyclonal anti-A2LD1 (Proteintech, 23280-1-AP, 1:500),<br>anti-rabbit IgG, HRP-linked antibody (7074S, Cell Signaling, WB 1:2000),<br>anti-mouse IgG, HRP-linked antibody (7076S, Cell Signaling, WB 1:2000).
Validation	The antibodies applied in this study are the widely-used clones in this field. We selected these antibodies which have been validated by the manufacturer and all information is available at the manufacturer website.

Eukaryotic cell lines

Policy information about cell lines

Cell line source(s)	HEK293T, HeLa, and A549 cell lines were purchased from the American Type Culture Collection (ATCC). 293TN cells were purchased from System Bioscience.
Authentication	Cell lines were authenticated by the supplier using Short Tandem Repeat (STR) profiling analysis.
Mycoplasma contamination	Cells were confirmed to be free of mycoplasma.
Commonly misidentified lines (See ICLAC register)	No commonly misidentified cell lines were used.

Animals and other organisms

Policy information about studies involving animals; ARRIVE guidelines recommended for reporting animal research

Laboratory animals	C57BL/6J mice were originally purchased from the Jackson Laboratory (Strain# 000664). 7 week old male and female mice were used. Mice were housed in a virus-free facility at 21 ± 1 °C with a controlled 12-hour light cycle (Individually Ventilated Caging System (GM500)). The animals have access to standard chow and water ad libitum. The relative humidity was controlled at 55% ± 10%.
Wild animals	The study did not involve wild animals.
Field-collected samples	The study did not involve filed-collected samples.
Ethics oversight	All mouse experiments were approved by the University of Chicago Institutional Animal Care and Use Committee.

Note that full information on the approval of the study protocol must also be provided in the manuscript.
