# Supplementary figures and images for: Quantitative sequencing using BID-seq uncovers abundant pseudouridines in mammalian mRNA at base resolution
Source: Nat Biotechnol. 2022 Oct 27;41(3):344–54. doi: 10.1038/s41587-022-01505-w (PMC10017504; doi:10.1038/s41587-022-01505-w)

Supplementary Fig. 7h

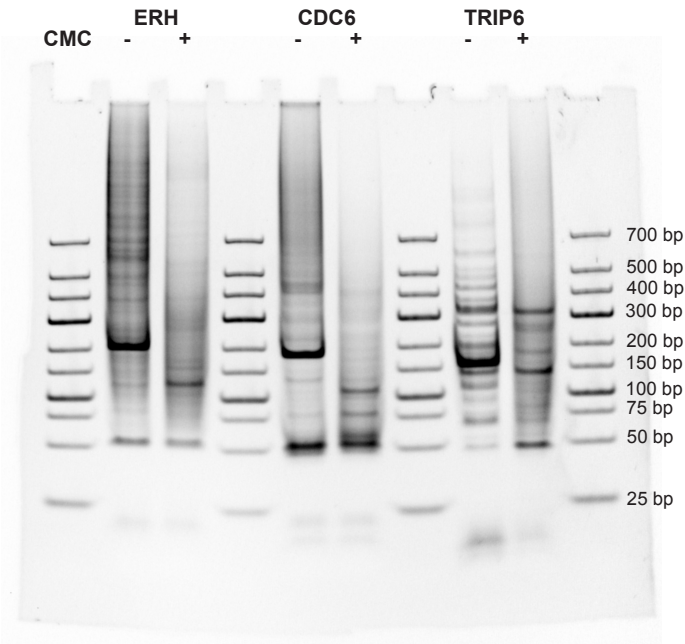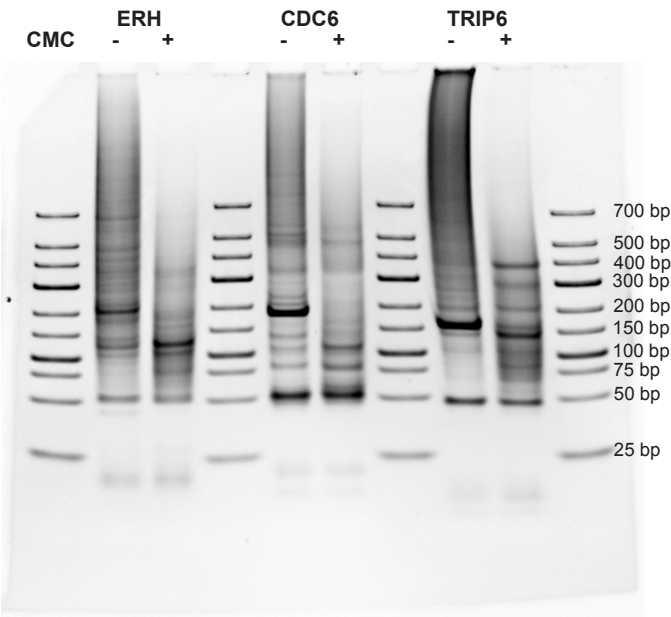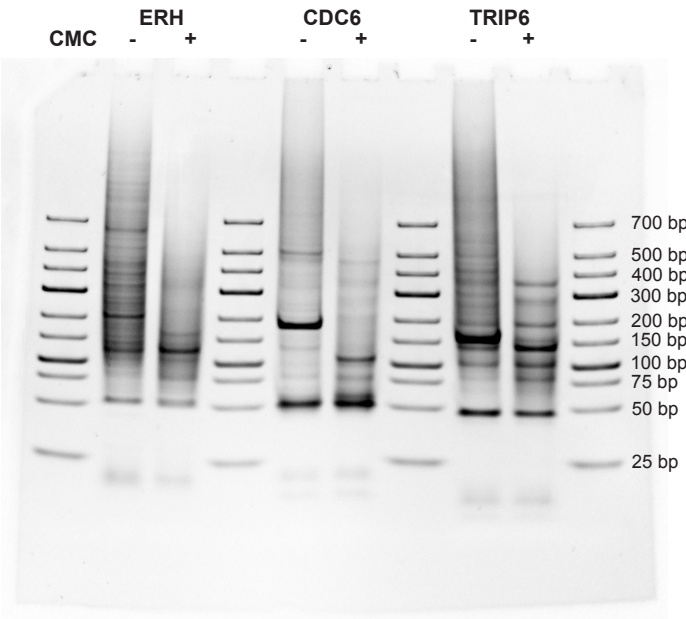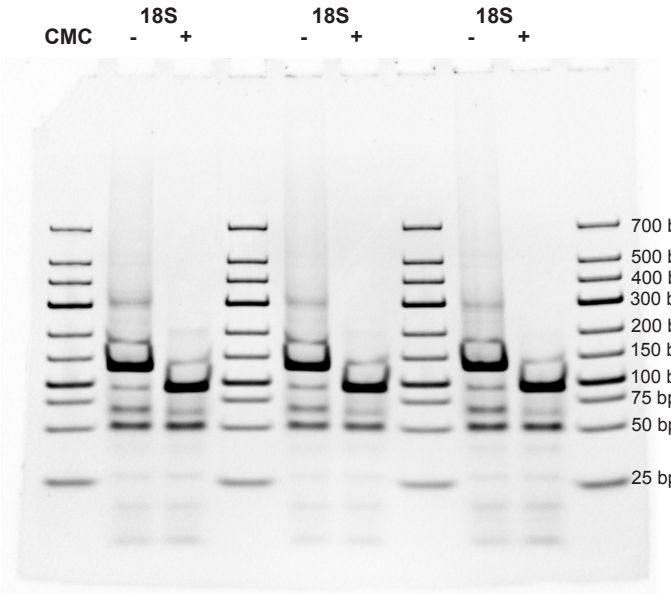

Supplement: Supplementary file 4 — Unprocessed gels for Supplementary Fig. 7h. [file 41587_2022_1505_MOESM4_ESM.pdf]

Supplementary Fig. 8d and 8e

Supp Fig 8d

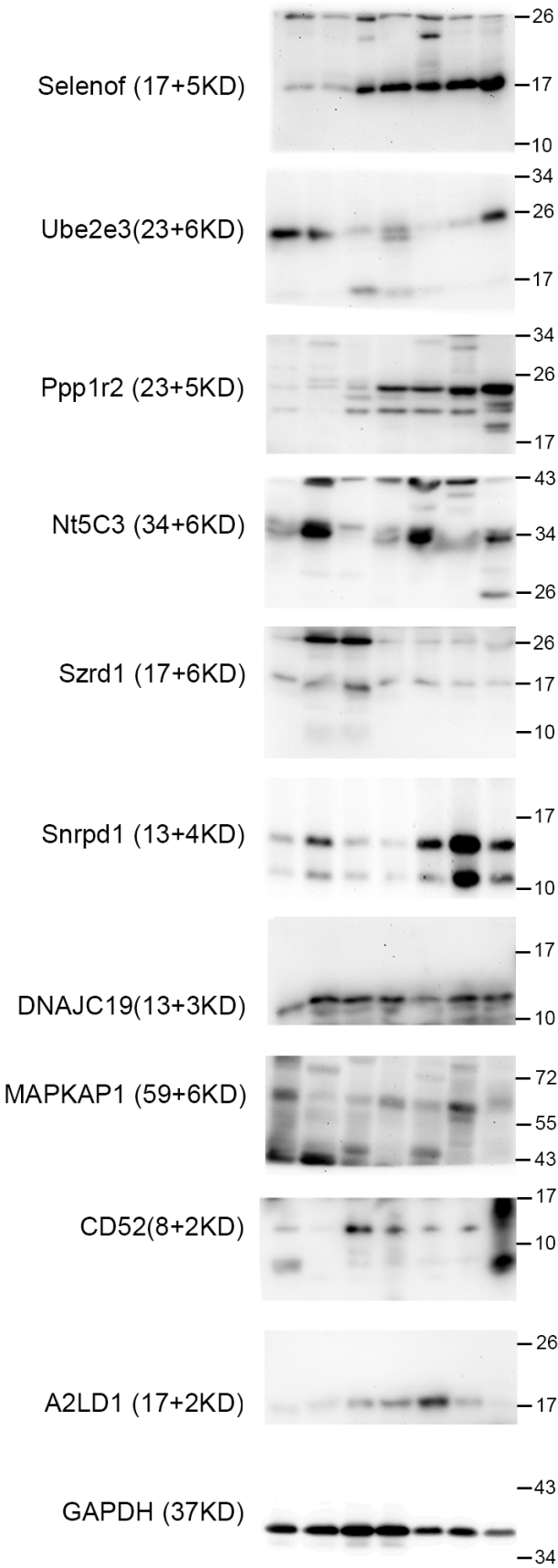

Supp Fig 8e

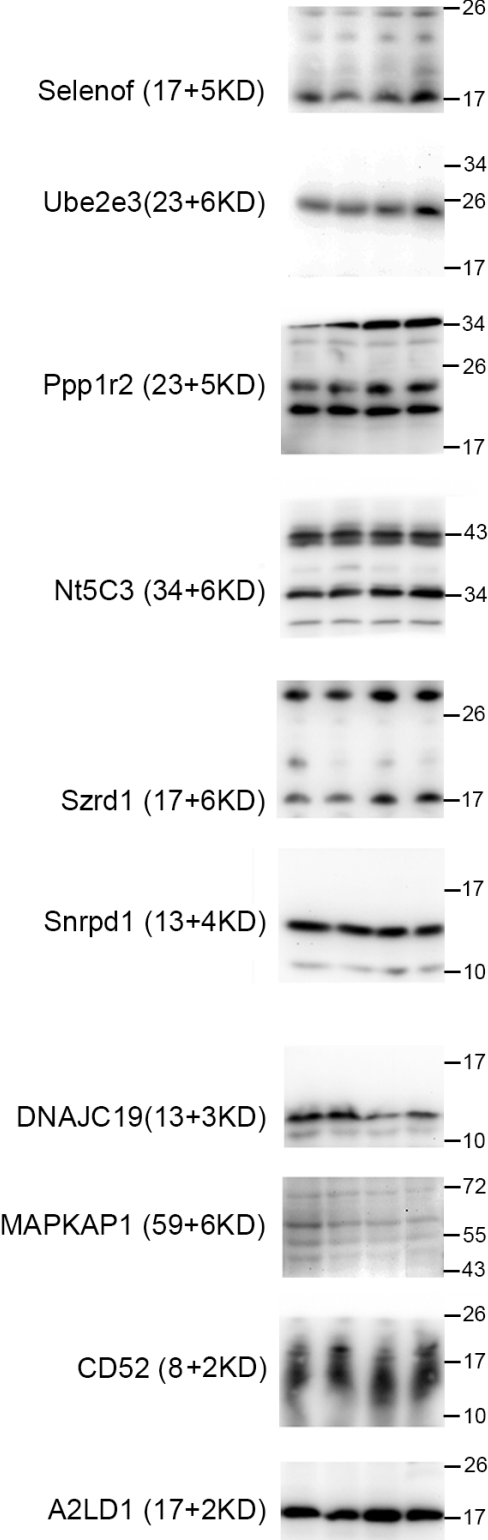

Supplement: Supplementary file 5 — Unprocessed blots for Supplementary Fig. 8d,e. [file 41587_2022_1505_MOESM5_ESM.pdf]

Fig. 6c

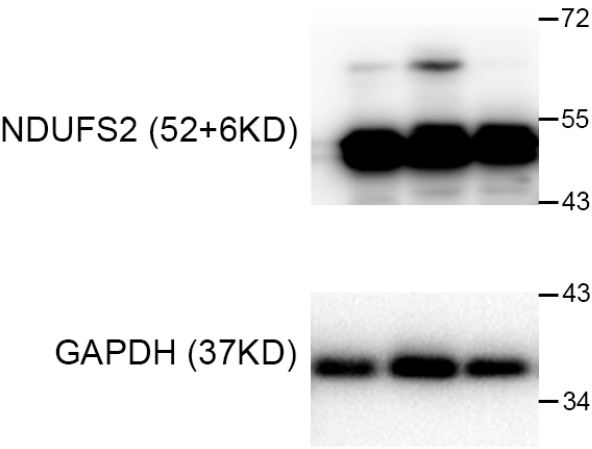

Supplement: Source Data Fig. 6 — Unprocessed blots for Fig. 6c. [file 41587_2022_1505_MOESM20_ESM.pdf]
